# Anesthetic Strategy, Functional Outcomes, and Infectious Complications After Mechanical Thrombectomy for Acute Ischemic Stroke

**DOI:** 10.3390/jcm15134993

**Published:** 2026-06-26

**Authors:** Aleksander Dębiec, Andrzej Michałowski, Katarzyna Boniecka, Julia Winnicka, Bartosz Rustecki, Piotr Zięcina, Jerzy Narloch, Piotr Piasecki, Adam Stępień, Jacek Staszewski

**Affiliations:** 1 Clinic of Neurology, Military Institute of Medicine—National Research Institute, 04-141 Warsaw, Polandjstaszewski@wim.mil.pl (J.S.); 2 Department of Anesthesiology and Intensive Care, Military Institute of Medicine—National Research Institute, 04-141 Warsaw, Poland; 3 Faculty of Medicine, University of Warsaw, 02-089 Warsaw, Poland; 4 Department of Radiology, Military Institute of Medicine—National Research Institute, 04-141 Warsaw, Poland

**Keywords:** acute ischemic stroke, mechanical thrombectomy, general anesthesia, conscious sedation, infectious complications, pneumonia, extubation, functional outcome

## Abstract

**Background/Objectives**: The optimal anesthetic strategy during mechanical thrombectomy (MT) for acute ischemic stroke (AIS) remains debated. Although randomized trials suggest broadly comparable outcomes between general anesthesia (GA) and conscious sedation (CS), real-world data may be influenced by baseline severity, airway management, and postprocedural complications. We evaluated associations between anesthetic strategy, functional outcomes, mortality, and infectious and hemorrhagic complications after MT. **Methods**: This retrospective observational study included 257 consecutive adults with AIS treated with MT at a single comprehensive stroke center. Patients were managed under CS or GA according to clinical and procedural considerations. Outcomes, mortality, infectious and hemorrhagic complications were compared between groups. Multivariable logistic regression assessed associations with 90-day functional independence and mortality, adjusting for baseline and procedural factors. In an exploratory GA subgroup analysis, outcomes were compared according to extubation timing, defined as early (≤6 h) or delayed (>6 h). **Results**: Of 257 patients, 155 (60.3%) underwent MT under CS and 102 (39.7%) under GA. GA-treated patients had higher baseline NIHSS scores and worse unadjusted functional outcomes throughout follow-up. After adjustment, GA remained associated with higher 90-day mortality (OR 4.39, 95% CI 1.50–12.84; *p* = 0.007) and lower odds of 90-day functional independence (OR 0.29, 95% CI 0.10–0.82; *p* = 0.020). Pneumonia was more frequent with GA (49.0% vs. 26.5%; *p* < 0.001), although attenuated in adjusted analyses. Delayed extubation was associated with worse outcomes, higher pneumonia rates, and more frequent symptomatic intracranial hemorrhage. **Conclusions**: GA was associated with worse functional outcomes and higher mortality after MT, but residual confounding and differences in baseline stroke severity likely contributed to these associations. Pneumonia and hemorrhagic complications may identify patients at increased risk of poor outcome, especially when extubation is delayed. Findings require prospective confirmation.

## 1. Introduction

Mechanical thrombectomy (MT) has become a cornerstone of treatment for acute ischemic stroke (AIS) caused by large-vessel occlusion (LVO), substantially improving functional outcomes [1,2,3]. The benefit of endovascular therapy has been demonstrated both in early time windows and, with advanced imaging selection, in extended time windows up to 24 h after symptom onset [4,5]. Despite these advances, clinical outcomes after MT remain strongly influenced not only by baseline neurological deficit, infarct volume, time to reperfusion, and the recanalization success, but also by local and center specific workflow, procedural strategy, and periprocedural management in real-world practice [6,7].

One unresolved issue in the periprocedural management of patients undergoing MT is the optimal anesthetic strategy. General anesthesia (GA) may provide airway protection, reduce patient movement, improve procedural conditions, and facilitate treatment in agitated or clinically unstable patients. Conversely, conscious sedation (CS) may avoid intubation-related delays, hypotension, and ventilator-associated complications, while allowing continuous neurological assessment during the procedure [8,9]. Early observational studies and meta-analyses suggested worse outcomes among patients treated under GA; however, these findings may have been substantially affected by confounding by indication, as patients selected for GA are often more severely affected at baseline [10,11].

Randomized trials comparing GA with conscious or procedural sedation have provided more balanced evidence. Trials such as SIESTA, AnStroke, GOLIATH, CANVAS, GASS, AMETIS, and SEGA generally found no clear disadvantage of GA with respect to 90-day functional outcome or mortality when anesthesia was delivered according to standardized protocols [12,13,14,15,16,17,18]. Some randomized data even suggest comparable or improved reperfusion and functional outcomes under GA in selected settings. Nevertheless, the translation of these trial findings into routine clinical practice remains challenging, because current ESO/ESMINT and AHA/ASA guidelines do not support a uniform anesthetic strategy for all patients undergoing MT. Real-world anesthesia decisions are individualized and frequently driven by clinical instability, impaired consciousness, aspiration risk, agitation, or anticipated procedural difficulty. Therefore, routine practice usually differs from selected patients and top centers included in clinical trials [8,11].

Beyond functional outcome, infectious complications may represent an underappreciated consequence of anesthetic management during MT. Endotracheal intubation, mechanical ventilation, impaired airway reflexes, and prolonged sedation may increase the risk of pulmonary infection, particularly pneumonia, through secretion retention, impaired cough, reduced mucociliary clearance, and microaspiration [19,20,21]. However, pulmonary complications have been inconsistently reported in randomized trials comparing anesthesia strategies during MT, and only recently has extubation timing after thrombectomy under GA been specifically addressed in randomized research [17,22,23]. This leaves an important gap in understanding whether worse outcomes observed in some patients treated under GA reflect the anesthetic strategy itself, baseline stroke severity, periprocedural airway management, complications related to prolonged ventilation, or blood pressure variability related to anesthesia strategy.

In this study, we evaluated the association between anesthetic strategy and clinical outcomes in a real-world cohort of patients undergoing MT for AIS. We compared patients treated under GA and CS with respect to functional outcomes, mortality, reperfusion success, and infectious complications. In addition, we performed adjusted analyses accounting for baseline stroke severity, infarct extent, treatment times, reperfusion success, and procedural variables. Finally, within the GA subgroup, we explored whether early versus delayed extubation was associated with infectious complications and long-term clinical outcomes.

We hypothesized that GA, compared with CS, would be associated with lower 90-day functional independence and higher rates of mortality and infectious complications, and that delayed extubation among patients treated under GA would identify a subgroup at increased risk of pneumonia and poor clinical outcome.

## 2. Materials and Methods

### 2.1. Study Design and Population

This retrospective observational study included consecutive adult patients with acute ischemic stroke who underwent MT at a single comprehensive stroke center admitted between January 2021 and December 2023. Patients treated during the study period were eligible if LVO was confirmed on baseline imaging and MT was performed within the standard treatment window of up to 6 h from symptom onset, according to current national and European guidelines [3,24]. Patients were excluded if key clinical or outcome data were unavailable.

All patients received care in the Neurointensive Stroke Care Unit and were treated according to AHA/ASA and ESO-ESMINT guidelines [2,3]. The study is reported in accordance with the STROBE statement [25].

### 2.2. Anesthesia Strategy

CS was defined as procedural analgesia and sedation without endotracheal intubation, whereas GA involved endotracheal intubation and mechanical ventilation. In the absence of a uniform recommendation, the choice of anesthesia strategy—CS or GA—was made jointly by the treating neurointerventionalist, neurologist, and anesthesiologist according to local practice and individualized assessment of neurological status, cooperation, level of consciousness, airway protection, aspiration risk, respiratory and hemodynamic stability, and anticipated procedural complexity. CS was generally preferred in cooperative patients able to protect their airway, whereas GA was selected in patients with reduced consciousness, agitation, inability to cooperate, active vomiting or aspiration risk, respiratory compromise, need for airway control, or anticipated technically complex procedures. This approach was consistent with available recommendations supporting the individualized selection of an anesthetic technique during MT rather than routine use of either GA or CS in all patients [3,26].

Patients were analyzed according to the initially intended anesthesia strategy at the start of the procedure. Conversion from CS to GA during MT occurred in 19 patients, and these cases were retained in the CS group for all analyses, in accordance with the intention-to-treat principle. This approach was used to reduce indication bias related to intraprocedural deterioration or technical difficulties requiring conversion.

### 2.3. Data Collection

The baseline demographic and clinical variables included age, sex, pre-stroke functional status assessed using the modified Rankin Scale (mRS) [27,28], stroke severity measured by the National Institutes of Health Stroke Scale (NIHSS) at admission [29], and infarct extent assessed using the Alberta Stroke Program Early CT Score (ASPECTS) [30]. Vascular territory was categorized as anterior or posterior circulation. Although detailed information on the specific occluded vessel was available, vessel location was classified as anterior versus posterior circulation for the main analyses. This approach was chosen to avoid sparse data across individual vessel categories, reduce model complexity, and reflect clinically meaningful differences in stroke presentation and prognosis between anterior and posterior circulation strokes. We provided detailed data about occluded artery in Supplementary Table S1.

Information on intravenous thrombolysis (rtPA), onset-to-groin puncture time (OTG), onset-to-reperfusion time (OTTICI), number of thrombectomy passes, reperfusion status, and pre-stroke medications was collected. Successful reperfusion was defined as TICI 2b–3. Pre-stroke pharmacotherapy included antiplatelet therapy (aspirin, clopidogrel, dual antiplatelet therapy, or other antiplatelet agents), anticoagulation (non-vitamin K antagonist oral anticoagulants [NOAC], vitamin K antagonists [VKA], low-molecular-weight heparin [LMWH]), and statin therapy.

Periprocedural blood pressure parameters were collected retrospectively from anesthesia records. Systolic (SBP) and diastolic (DBP) blood pressure values were recorded at the beginning of anesthesia, and at the end of anesthesia. Proper blood pressure control was defined as SBP 140–180 mmHg with DBP ≤ 105 mmHg according to AHA/ASA and European Stroke Organisation guidelines and, for the purposes of our study, this parameter was collected as a binary variable and labeled as excellent blood pressure control (at both the beginning and end of anesthesia) [2,31].

### 2.4. Outcome Measures

The primary outcome was functional independence at 90 days, defined as a modified Rankin Scale (mRS) score of 0–2. Key secondary outcomes included 90-day mortality, pneumonia during hospitalization, and any infectious complication during hospitalization. Additional secondary outcomes included mRS at 30 days, 90 days, and 12 months, functional independence at 30 days and 12 months, in-hospital mortality, 30-day mortality, 12-month mortality. Additional safety outcomes were defined as the rate of pneumonia and occurrence of symptomatic intracranial hemorrhage (sICH).

Early neurological outcome was assessed using the NIHSS at 24 h and at hospital discharge. Follow-up CT was performed at 24 h to assess infarct extent using ASPECTS, and detect hemorrhagic transformation. ASPECTS assessed on follow-up was analyzed descriptively using available cases only and was not included in the main multivariable models. Symptomatic intracranial hemorrhage was defined as hemorrhagic transformation on follow-up CT associated with neurological deterioration of ≥4 points on the NIHSS or death attributable to the hemorrhage. Hemorrhagic transformation without clinical deterioration was classified as asymptomatic intracranial hemorrhage [32].

Functional outcome was assessed using the mRS at discharge, 30 days, 90 days, and 12 months. When an in-person visit was not possible, outcomes were obtained via a structured telephone interview; if the patient could not be reached, the family/caregiver was contacted to ascertain functional status and vital status. Functional independence was defined as mRS 0–2 at 30 days, 90 days and 12 months.

Mortality was analyzed cumulatively as in-hospital mortality, 30-day mortality, 90-day mortality, and 12-month mortality. In addition, an exploratory interval-specific mortality analysis was performed. Deaths were categorized as occurring within 30 days, between 31 and 90 days, or between 91 days and 12 months.

Survival was evaluated using Kaplan–Meier curves, with time-to-event defined according to available follow-up duration and death during follow-up as the event of interest. Survival curves were compared between anesthesia groups using the log-rank test.

Infectious complications were recorded during hospitalization and included pneumonia and urinary tract infection. Pneumonia was defined as a clinically diagnosed lower respiratory tract infection supported by new or progressive infiltrates on chest imaging (X-ray or CT scan) and compatible clinical or laboratory features, including fever, leukocytosis or leukopenia, purulent sputum or increased respiratory secretions, respiratory symptoms, abnormal auscultatory findings, or worsening oxygenation, consistent with CDC/NHSN and stroke-associated pneumonia criteria [33,34]. Urinary tract infection was defined as a symptomatic infection diagnosed during hospitalization and supported by clinical features and/or fever together with urinalysis or urine culture findings, according to CDC/NHSN criteria [35]. Any infectious complication was defined as pneumonia and/or urinary tract infection. Infectious complications were identified retrospectively from clinical records according to predefined clinical, radiological, and laboratory criteria, including documentation of suspected or confirmed pulmonary and urinary tract infection during hospitalization. Diagnosis was not adjudicated by an independent blinded committee. Pneumonia and suspected ventilator-associated pneumonia, and urinary tract infections were managed according to local neurointensive care practice. Treatment generally was managed according to hospital antibiotic susceptibility profile and included empiric antibiotic therapy when clinically indicated, adjustment of antimicrobial therapy according to microbiological results when available, respiratory physiotherapy, airway clearance, oxygen therapy, and ventilatory support depending on respiratory and neurological status. Antibiotic regimens, microbiological sampling, and the duration of treatment were not standardized for study purposes and were determined by the treating physicians. Ventilator-associated pneumonia was not analyzed as a separate independently adjudicated endpoint. Therefore, pneumonia was reported as an overall in-hospital pulmonary infectious complication.

### 2.5. General Anesthesia Subgroup and Extubation Timing

An exploratory subgroup analysis was performed among patients treated under GA. Patients were categorized according to extubation timing as early extubation, defined as extubation within 6 h, or delayed extubation, defined as extubation after 6 h. Patients with missing or non-informative extubation data were excluded from this subgroup analysis. Baseline characteristics, procedural variables, pre-stroke pharmacotherapy, infectious complications, mortality, and functional outcomes were compared between early and delayed extubation groups.

The decision to extubate early or to continue mechanical ventilation was made by the treating anesthesiology and neurointensive care team according to clinical status. Delayed extubation was generally considered in patients with impaired consciousness, insufficient airway protection, persistent respiratory instability, suspected or documented aspiration risk, need for continued ventilatory support, hemodynamic instability, or postprocedural neurological deterioration, including hemorrhagic complications. Extubation timing was not protocolized for study purposes and therefore reflects routine clinical decision-making. For this reason, the early versus delayed extubation comparison was considered exploratory.

### 2.6. Statistical Analysis

There was no loss to follow-up or missing data for the clinical secondary outcome measures, except those related to deceased patients. The Shapiro–Wilk test was used to assess the normality of the variables. Continuous and ordinal variables are presented as median with interquartile range (IQR) and were compared using the Mann–Whitney U test. Categorical variables are presented as counts and percentages and were compared using the χ^2^ test or Fisher exact test, as appropriate. The overall distribution of mRS at 30 days, 90 days and 12 months was compared between groups (shift in disability levels) using the Wilcoxon–Mann–Whitney test and presented graphically as stacked proportional mRS shift plots.

Absolute risk differences were calculated for selected binary outcomes as the difference in event proportions between the general anesthesia and conscious sedation groups. For adverse outcomes, the number needed to harm was calculated as the reciprocal of the absolute risk increase and rounded up to the nearest whole number. Because of the observational study design, absolute risk differences and numbers needed to harm were interpreted as descriptive unadjusted measures rather than causal effect estimates.

To further address baseline imbalance between anesthesia groups, an additional propensity score sensitivity analysis was performed. The propensity score for treatment under GA was estimated using a logistic regression model including clinically relevant variables available before or at the time of anesthesia selection: age (per decade), sex, pre-stroke mRS, baseline NIHSS score, baseline ASPECTS, vascular territory (anterior vs. posterior), intravenous thrombolysis, and major vascular risk factors (AF, hypertension, diabetes mellitus, current smoking, coronary artery disease, hyperlipidemia, previous TIA or Stroke). Patients treated under GA and CS were matched using 1:1 nearest-neighbor matching without replacement with a caliper of 0.2 standard deviations of the logit of the propensity score. The covariate balance before and after matching was assessed using standardized mean differences, with values below 0.10 considered to indicate acceptable balance. Binary outcomes in the matched cohort were compared using McNemar’s test. The covariate balance before and after matching was assessed using standardized mean differences. In the matched cohort, 90-day functional independence, 90-day mortality, pneumonia, and any infectious complication were compared between anesthesia groups.

To assess the robustness of our findings and determine whether specific baseline characteristics or treatment-related variables are associated with anesthesia strategy and 90-day functional independence, 90-day mortality, and infectious complications, we conducted sensitivity analyses using multivariate logistic regression adjusting for prespecified covariates including age (per decade), baseline NIHSS score on admission, baseline ASPECTS on admission, vascular territory (anterior vs. posterior circulation), bridging rtPA, onset-to-reperfusion time, number of thrombectomy passes, successful reperfusion, and blood pressure control on the beginning and in the end of anesthesia evidenced by excellent blood pressure control. Covariates for multivariable adjustment were selected based on clinical relevance and presumed relationships with both anesthetic strategy and clinical outcomes. Additional exploratory analyses were performed using separate models adjusted for statistically significant covariates that were not prespecified. The results are reported as odds ratios (ORs) with 95% confidence intervals (CI). For each multivariable logistic regression model, the number of complete cases, number of outcome events, and events-per-variable ratio were calculated. The events-per-variable ratio was defined as the number of patients experiencing the modeled outcome divided by the number of covariates included in the model. Model discrimination was assessed using the area under the receiver operating characteristic curve. Model calibration was assessed using the Hosmer–Lemeshow goodness-of-fit test. Multicollinearity among covariates was assessed using variance inflation factors. Models additionally including non-prespecified covariates were considered exploratory because of sparse events in some strata and the potential for unstable estimates.

Because several secondary and exploratory outcomes were analyzed, the possibility of multiplicity was considered. No formal correction for multiple testing was applied, as the study was observational and the analyses were exploratory and hypothesis-generating rather than confirmatory. Therefore, *p* values from secondary and exploratory analyses should be interpreted as nominal.

We have conducted this study in accordance with the Declaration of Helsinki. The electronic database was decoded, and the patient identification data was scrambled to ensure confidentiality; informed consent was thus exempted. The evaluation of all the imaging studies was blinded from the clinical data. The studies involving human participants were reviewed and approved by Ethics Committee of Military Institute of Medicine in Warsaw decision number 34/WIM/2020. Written informed consent for participation was not required for this study, in accordance with the national legislation and the institutional requirements.

The statistical analyses were performed with the PQStat software (v1.8.6.122, Poznań, Poland, 2024).

## 3. Results

### 3.1. Baseline and Procedural Characteristics

A total of 257 consecutive patients with AIS treated with MT between January 2021 and December 2023 were included in the analysis. Of these, 155 patients (60.3%) underwent the procedure under CS while 102 patients (39.7%) were treated under GA. (2021: 91 patients, CS = 56 [61.5%], GA = 35 [38.5%]; 2022: 76 patients, CS = 49 [64.5%], GA = 27 [35.5%]; 2023: 90 patients, CS = 50 [55.6%], GA = 40 [44.4%]). The characteristics of our cohort were summarized in Table 1.

#### 3.1.1. Demographics and Stroke Severity

The median age of the overall cohort was 72 years (IQR 64–82) and did not differ between groups. Women accounted for 54.9% of the study population, with a comparable sex distribution between CS and GA groups. Baseline functional status was comparable in both groups, with a median pre-stroke mRS of 0.

Patients treated under GA presented with significantly more severe neurological deficits on admission, reflected by a higher median NIHSS score compared with CS-treated patients (17 [IQR 14–21] vs. 14 [IQR 8–18]; *p* < 0.001). The baseline infarct volume, assessed using ASPECTS, was similar between groups.

#### 3.1.2. Stroke Localization and Acute Treatment

Occlusions in the anterior circulation predominated in the overall cohort (89.5%), but were significantly more frequent in the CS group, whereas posterior circulation strokes were more common among patients treated under GA (*p* = 0.009) [detailed data on the affected vessel are provided in Supplementary Table S1]. The use of rtPA did not differ significantly between groups.

#### 3.1.3. Comorbidities

The prevalence of vascular risk factors, including atrial fibrillation, hypertension, diabetes mellitus, smoking, coronary artery disease, hyperlipidemia, and prior TIA or stroke, was high but balanced between groups, with no statistically significant differences.

#### 3.1.4. Pre-Stroke Antithrombotic Therapy

Pre-stroke antiplatelet therapy was used in 18.3% of patients overall and did not differ between CS and GA groups. Similarly, the use of aspirin, clopidogrel, dual antiplatelet therapy, or other antiplatelet agents was comparable between groups. Anticoagulation therapy prior to stroke was present in approximately one-fifth of patients, with similar distributions of NOACs, VKAs, and LMWH between groups. Pre-stroke statin use was also balanced.

#### 3.1.5. Procedural Characteristics

Median onset-to-groin and onset-to-TICI times were similar in both groups. However, patients treated under GA required a significantly higher number of thrombectomy passes compared with those treated under CS (*p* = 0.002). Despite this difference, the rate of successful reperfusion (TICI 2b–3) was high overall (84.4%) and did not differ significantly between groups.

#### 3.1.6. Periprocedural Blood Pressure Management

At the beginning of anesthesia, the median SBP was 148.5 mmHg (IQR 133.0–166.0) in the CS group and 151.0 mmHg (IQR 134.5–177.5) in the GA group (*p* = 0.309), whereas the median DBP was 81.0 mmHg (IQR 74.0–93.3) and 86.0 mmHg (IQR 76.5–100.0), respectively (*p* = 0.162). At the end of anesthesia, the median SBP was 140.0 mmHg (IQR 130.0–157.0) in the CS group and 140.0 mmHg (IQR 125.0–174.0) in the GA group (*p* = 0.701), whereas the median DBP was 80.0 mmHg (IQR 70.0–90.0) and 80.0 mmHg (IQR 70.0–95.0), respectively (*p* = 0.661) (Table 1). Excellent blood pressure control at the beginning of anesthesia was achieved in 52.1% and did not differ significantly between patients treated under CS and GA (55.4% vs. 47.0%; *p* = 0.284). Similarly, excellent blood pressure control at the end of anesthesia was achieved in 44.3%, with no significant difference between anesthesia groups (CS vs. GA 46.3% vs. 40.9%; *p* = 0.488).

### 3.2. Clinical Outcomes

#### 3.2.1. Early Neurological and Imaging Outcomes

Neurological status at 24 h differed significantly between anesthesia strategies. Patients treated under CS had a markedly lower NIHSS score at 24 h compared with those treated under GA (median 8 [4–14] vs. 18 [12–22], *p* < 0.001).

Follow-up imaging at 24 h demonstrated a larger infarct extent in patients treated under GA. Median ASPECTS at 24 h was lower in the GA group compared with the CS group (6 [3–7] vs. 7 [5–8], *p* = 0.005). ASPECTS at 24 h was available for a subset of patients (Table 2). Hemorrhagic transformation at 24 h was numerically more frequent in patients treated under GA (GA vs. CS: 42.2% vs. 34.2%; *p* = 0.197). Symptomatic hemorrhagic transformation was more frequent in the GA group (GA vs. CS: 17.6% vs. 7.1%; *p* = 0.009), whereas asymptomatic hemorrhagic transformation did not differ between groups (GA vs. CS: 24.5% vs. 27.1%; *p* = 0.644).

#### 3.2.2. Neurological Status and Functional Outcome

At hospital discharge, patients treated under CS showed better neurological and functional outcomes. Median NIHSS at discharge was lower in the CS group compared with GA (4 [1–9] vs. 9 [4–16], *p* < 0.001). Similarly, functional status at discharge assessed by the modified Rankin Scale was more favorable in patients treated under CS (median mRS 4 [2–5] vs. 5 [4–6], *p* < 0.001).

This difference persisted during follow-up. At 30 days, patients in the CS group had a lower median mRS score compared with the GA group (3 [1–5] vs. 5 [3–6], *p* < 0.001). At 90 days, functional outcomes remained significantly better in patients treated under CS (median mRS 3 [1–5] vs. 5 [3–6], *p* < 0.001).

Functional independence at 90 days, defined as mRS 0–2, was achieved more frequently in patients treated under CS than in those treated under GA (45.8% vs. 21.6%, *p* < 0.001).

Mortality rates were consistently higher in the GA group. In-hospital mortality was 12.9% in the CS group compared with 27.5% in the GA group (*p* = 0.003). Thirty-day mortality was also lower in patients treated under CS (14.2% vs. 28.4%, *p* = 0.005). At 90 days, mortality remained significantly lower in the CS group compared with GA (22.6% vs. 42.2%, *p* < 0.001).

At 12 months, functional outcome remained worse in the GA group, with a higher median mRS (5 [3–6] vs. 3 [1–6], *p* < 0.001), a lower functional independence rate (23.5% vs. 48.4%, *p* < 0.001), and a higher mortality (49.0% vs. 32.3%, *p* = 0.007) (Table 2).

**Figure 1:**
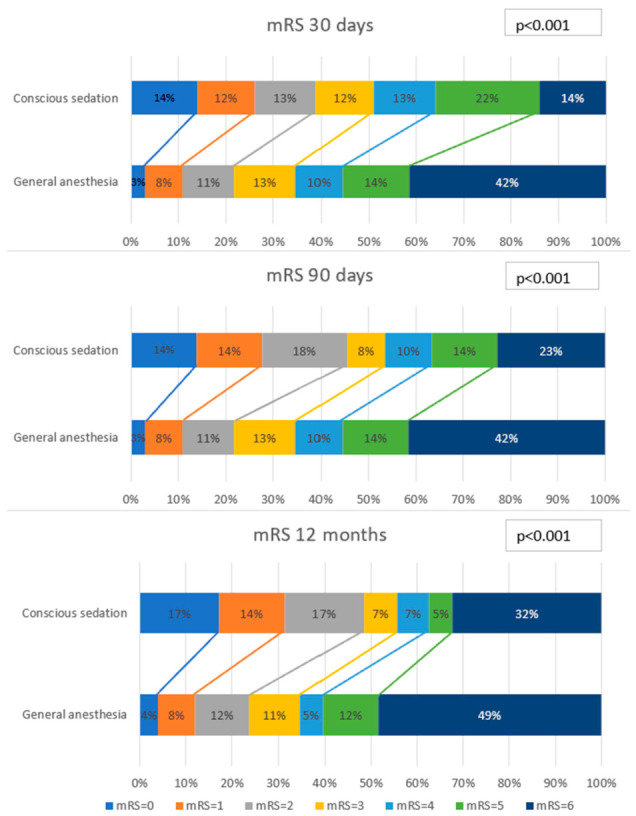
Unadjusted distribution of 30-day, 90-day and 12-month mRS scores for patients treated under CS vs. GA. Stacked bars show the percentage distribution of mRS scores from zero to six in patients treated under conscious sedation and general anesthesia. Differences in ordinal mRS distributions were assessed using the Wilcoxon–Mann–Whitney test. mRS, modified Rankin Scale.

In unadjusted ordinal mRS shift analyses, patients treated under GA showed a consistent shift toward worse functional outcomes compared with those treated under CS at all follow-up time points (Figure 1).

Mortality was primarily analyzed cumulatively at 30 days, 90 days, and 12 months. In an additional exploratory analysis, deaths were categorized according to the interval in which they occurred: within 30 days, between 31 and 90 days, and between 91 days and 12 months. Patients who died in an earlier interval were not included as events in subsequent intervals. The excess mortality associated with GA was most pronounced within the first 30 days after stroke (*p* = 0.005). Among patients who survived the first 30 days, mortality between days 31 and 90 showed a non-significant trend toward higher risk in the GA group, whereas mortality between 90 days and 12 months was comparable between groups (Table 3).

For selected clinically relevant binary outcomes, absolute risk differences were calculated to facilitate interpretation of the unadjusted group differences. Compared with CS, GA was associated with a lower rate of 90-day functional independence (21.6% vs. 45.8%; absolute risk difference, −24.2 percentage points). General anesthesia was also associated with higher unadjusted risks of 90-day mortality (42.2% vs. 22.6%; absolute risk difference, +19.6 percentage points; descriptive number needed to harm, 6), pneumonia (49.0% vs. 26.5%; absolute risk difference, +22.5 percentage points; descriptive number needed to harm, 5), and any infectious complication (49.0% vs. 28.4%; absolute risk difference, +20.6 percentage points; descriptive number needed to harm, 5). These estimates were interpreted as descriptive unadjusted measures and not as causal effects.

### 3.3. Adjusted Analyses of Functional Outcomes

**Figure 2:**
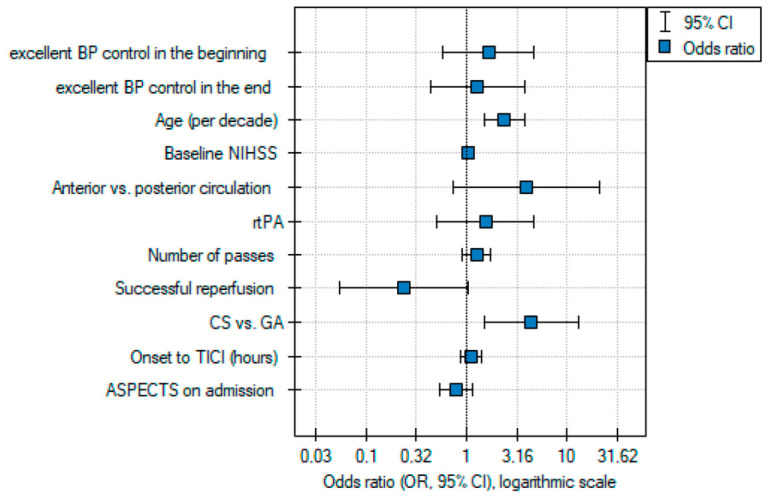
Adjusted analysis of 90-day mortality.

**Figure 3:**
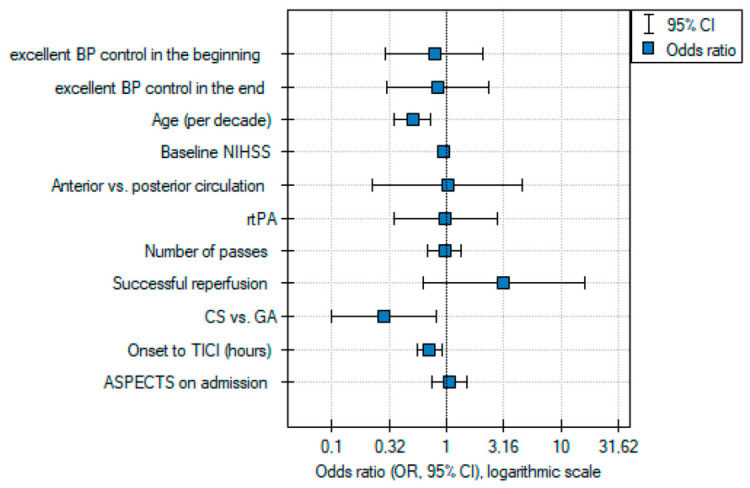
Adjusted analysis for functional independence at 90 days.

In multivariable logistic regression analyses adjusted for excellent blood pressure control in the beginning and in the end of anesthesia, age (per decade), baseline NIHSS score on admission, vascular territory (anterior vs. posterior circulation), bridging rtPA, number of passes, successful reperfusion (TICI 2b-3), anesthetic strategy (CS vs. GA), onset-to-TICI time (in hours) and baseline ASPECTS, anesthetic strategy remained independently associated with functional outcomes at 90 days. GA was associated with a significantly higher risk of death at 90 days compared with CS (adjusted OR 4.39, 95% CI 1.50–12.84; *p* = 0.007). Age (per decade) was also independently associated with a higher 90-day mortality (OR 2.37, 95% CI 1.49–3.77; *p* < 0.001). Successful reperfusion showed a strong protective trend for lower mortality but did not reach statistical significance (OR 0.24, 95% CI 0.05–1.02; *p* = 0.054) (Figure 2). GA was independently associated with lower odds of functional independence at 90 days (mRS 0–2) (adjusted OR 0.29, 95% CI 0.10–0.82; *p* = 0.02). Older age (OR 0.50 per decade, 95% CI 0.35–0.73; *p* < 0.001) and longer onset-to-TICI (in hours) (OR 0.71 per hour, 95% CI 0.55–0.91; *p* = 0.006) were associated with lower odds of functional independence at 90 days (Figure 3).

In an exploratory sensitivity analysis additionally adjusted for sICH general anesthesia remained independently associated with a higher 90-day mortality (OR 4.02, 95% CI 1.20–13.52; *p* = 0.024) and a lower odds of functional independence at 90 days (OR 0.33, 95% CI 0.11–0.95; *p* = 0.040). sICH was strongly associated with 90-day mortality (OR 94.72, 95% CI 5.72–1567.85; *p* = 0.001). However, the adjusted estimate for sICH should be interpreted cautiously. The very large odds ratio and wide confidence interval suggest limited precision, most likely related to the relatively low number of sICH events and sparse data in some model strata. Therefore, this result should not be viewed as a precise quantification of the independent effect of sICH. Rather, sICH should be interpreted as a strong marker of severe postprocedural neurological injury and poor prognosis in this cohort (Supplementary Figures S1 and S2).

### 3.4. Survival Analysis According to Anesthesia Type

**Figure 4:**
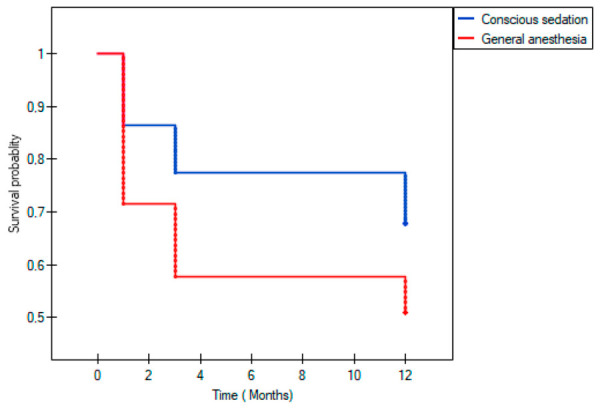
Kaplan–Meier survival analysis according to anesthesia strategy during 12 months of follow up.

Kaplan–Meier survival analysis showed early separation of survival curves within the first 3 months after stroke, with persistently worse survival among patients treated under general anesthesia throughout the 12-month follow-up period (log-rank *p* = 0.003) These findings were consistent across the predefined follow-up intervals and supported the unadjusted mortality differences observed in earlier analyses (Figure 4).

### 3.5. Infectious Complications

Infectious complications occurred in 36.6% of the overall cohort and were significantly more frequent among patients treated under GA compared with CS (49.0% vs. 28.4%, *p* = 0.001).

This difference was driven primarily by a higher incidence of pneumonia in the GA group (49.0% vs. 26.5%, *p* < 0.001), whereas the rate of urinary tract infection was similar between groups (17.6% vs. 18.1%, *p* = 0.93) (Table 4).

**Figure 5:**
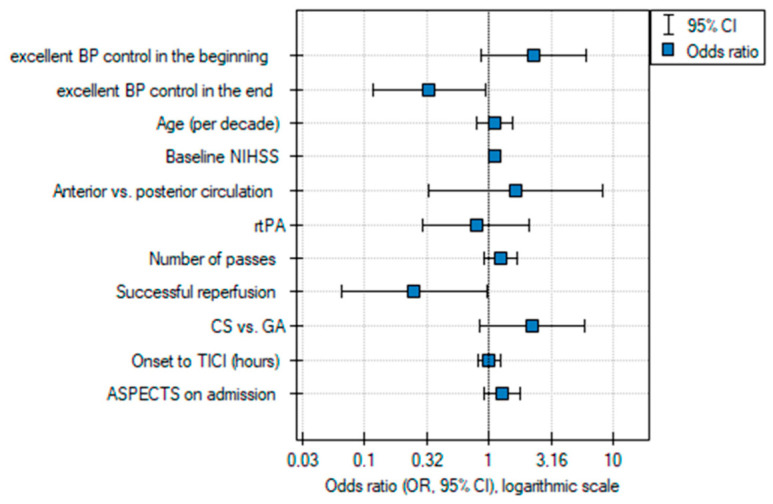
Adjusted analysis of risk of any infectious complication.

**Figure 6:**
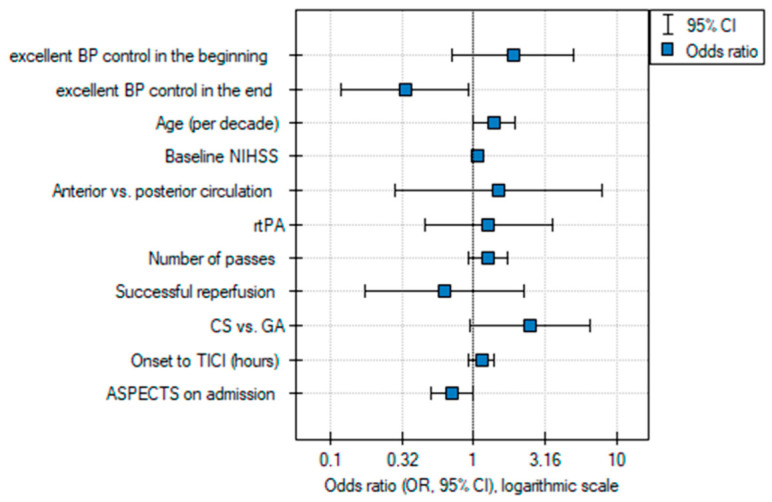
Adjusted analysis of risk of pneumonia.

In multivariable logistic regression models adjusted for excellent blood pressure control at the beginning and end of anesthesia, age per decade, baseline NIHSS score, vascular territory, bridging rtPA, number of thrombectomy passes, successful reperfusion, anesthetic strategy, onset-to-TICI time, and baseline ASPECTS, general anesthesia showed a consistent trend toward higher odds of any infectious complication (OR 2.21, 95% CI 0.84–5.83; *p* = 0.108) and pneumonia (OR 2.48, 95% CI 0.94–6.57; *p* = 0.068), although these associations did not reach statistical significance (Figure 5 and Figure 6). After additional adjustment for sICH, the trend remained visible but was attenuated and non-significant for both any infectious complication (OR 1.77, 95% CI 0.65–4.88; *p* = 0.267) and pneumonia (OR 2.08, 95% CI 0.76–5.74; *p* = 0.157). In these models, sICH was independently associated with both any infectious complication (OR 7.70, 95% CI 1.74–34.11; *p* = 0.007) and pneumonia (OR 5.60, 95% CI 1.30–24.12; *p* = 0.021), whereas excellent blood pressure control at the end of anesthesia was associated with a lower odds of any infectious complication (OR 0.29, 95% CI 0.10–0.87; *p* = 0.028) and pneumonia (OR 0.30, 95% CI 0.10–0.87; *p* = 0.027). These findings suggest a possible trend toward increased infectious risk among patients treated under GA, particularly for pneumonia, but these associations did not reach statistical significance after adjustment and should be interpreted cautiously. Therefore, these results were interpreted as exploratory.

Model stability diagnostics are presented in Supplementary Table S2. The main multivariable logistic regression models included 11 covariates. The complete-case sample ranged from 185 to 189 patients depending on the outcome model, with 55 to 69 outcome events. The events-per-variable ratios ranged from 5.0 to 6.3, indicating a limited number of events relative to the number of covariates. Therefore, adjusted estimates should be interpreted cautiously. Despite the limited events-per-variable ratios, the models showed acceptable to good discrimination, with areas under the receiver operating characteristic curve ranging from 0.773 to 0.852. Calibration assessment using the Hosmer–Lemeshow test showed no evidence of poor model fit for the main models. No relevant multicollinearity was observed among covariates (all VIF values < 2).

### 3.6. Propensity Score Matching Sensitivity Analysis

In the propensity score matching sensitivity analysis, 204 patients had complete data for all variables entered the propensity score model, including 83 patients treated under general anesthesia and 121 patients treated under conscious sedation. Using 1:1 nearest-neighbor matching without replacement with a caliper of 0.2179, 70 matched pairs were identified. Before matching, substantial imbalance was observed for the propensity score, baseline NIHSS, and vascular territory (anterior vs. posterior). The standardized mean difference for baseline NIHSS was 0.726, and the standardized mean difference for posterior circulation was 0.394. After matching, covariate balance improved but remained imperfect, particularly for baseline NIHSS and posterior circulation, with standardized mean differences of 0.264 and 0.270, respectively.

In the propensity score-matched cohort, 90-day functional independence was observed in 15/70 patients treated under general anesthesia and 26/70 patients treated under conscious sedation (21.4% vs. 37.1%; McNemar *p* = 0.052). Additionally, 90-day mortality occurred in 29/70 patients in the general anesthesia group and 16/70 patients in the conscious sedation group (41.4% vs. 22.9%; McNemar *p* = 0.029). Pneumonia occurred in 33/70 and 20/70 patients, respectively (47.1% vs. 28.6%; McNemar *p* = 0.053), whereas any infectious complication occurred in 30/70 and 19/70 patients, respectively (42.9% vs. 27.1%; McNemar *p* = 0.108).

Overall, the propensity score matching sensitivity analysis showed results directionally consistent with the primary analyses. Treatment under GA remained associated with lower rates of functional independence and higher rates of mortality and pneumonia in the matched cohort. These findings support the robustness of the observed associations but also confirm that baseline stroke severity and confounding by indication remain important considerations when interpreting the relationship between anesthetic strategy and outcome. Therefore, these results should be interpreted cautiously and do not establish causality.

The covariate balance before and after propensity score matching is presented in Supplementary Table S3 and Figure S3. The clinical outcomes in the propensity score-matched cohort are presented in Supplementary Table S4.

### 3.7. Differences Between Early and Delayed Extubation Group

Among patients treated under GA (*n* = 102), 55 were extubated early (<6 h) and 47 underwent late extubation (>6 h). Patients extubated later presented with more severe neurological deficits on admission, as reflected by higher baseline NIHSS scores (median 19 vs. 16, *p* = 0.017), and had larger infarct extent assessed by ASPECTS on admission (median 8.5 vs. 10, *p* = 0.006). Age, sex distribution, and the proportion of anterior versus posterior circulation strokes did not differ significantly between groups.

Procedural characteristics showed no significant differences in onset-to-groin puncture or onset-to-reperfusion times. However, patients in the late extubation group achieved successful reperfusion less frequently compared with those extubated early (70.2% vs. 89.1%, *p* = 0.017), in the event of no difference in the number of passes. Delayed extubation was associated with a higher rate of hemorrhagic transformation at 24 h compared with early extubation (57.4% vs. 29.1%; *p* = 0.004). This difference was driven primarily by sICH, which was more frequent in the delayed extubation group (34.0% vs. 3.6%; *p* < 0.001), whereas the rate of aICH was similar between groups (23.4% vs. 25.5%; *p* = 0.810).

Among patients treated under GA, blood pressure parameters did not differ significantly between early and delayed extubation groups, and a rate of excellence blood pressure control was similar. Excellent blood pressure control at the beginning of anesthesia was observed in 42.9% of early-extubated patients and 50.0% of delayed-extubated patients (*p* = 0.558), whereas excellent blood pressure control at the end of anesthesia was observed in 37.1% and 43.8%, respectively (*p* = 0.582).

Clinical outcomes were consistently worse in the late extubation group. These patients exhibited higher NIHSS scores at discharge and worse functional status at discharge, 30 days, 90 days, and 12 months. The mortality rates at 30 days, 90 days, and 12 months were significantly higher among patients extubated after 6 h. Although functional independence at 90 days was numerically lower in the late extubation group, this difference did not reach statistical significance.

Infectious complications were markedly more frequent among patients with delayed extubation. Any infectious complication occurred in 31/47 patients (66.0%) in the delayed extubation group compared with 19/55 patients (34.5%) in the early extubation group (*p* = 0.002). This difference was primarily driven by pneumonia, which was observed in 33/47 patients (70.2%) with delayed extubation and 17/55 patients (30.9%) with early extubation (*p* < 0.001). In contrast, urinary tract infection rates did not differ significantly between groups (21.3% vs. 14.5%; *p* = 0.374), suggesting that the excess infectious burden in the delayed extubation group was mainly related to pulmonary complications (Supplementary Table S5). Because extubation timing was determined by clinical status rather than random allocation, substantial baseline and postprocedural differences between early and delayed extubation groups should be considered when interpreting this subgroup analysis. Patients with delayed extubation had more severe neurological deficits on admission, lower baseline ASPECTS, lower rates of successful reperfusion, and more frequent symptomatic intracranial hemorrhage. These differences suggest that delayed extubation identified a clinically more severe subgroup with greater neurological injury and higher medical instability. Therefore, the associations between delayed extubation and worse functional outcomes, mortality, pneumonia, and symptomatic intracranial hemorrhage should be interpreted as exploratory. Delayed extubation may contribute to pulmonary complications through prolonged ventilation and impaired airway clearance, but it may also represent a marker of impaired consciousness, insufficient airway protection, respiratory instability, aspiration risk, or neurological deterioration.

## 4. Discussion

In this real-world cohort of patients undergoing MT for AIS, several clinically relevant findings emerged. Patients treated under GA presented with more severe neurological deficits at admission and experienced worse unadjusted neurological and functional outcomes compared with those managed under CS. After adjustment for baseline stroke severity, infarct extent, vascular territory, reperfusion success, and procedural variables, GA remained associated with lower odds of functional independence and higher mortality at 90 days. GA was also associated with a higher rate of infectious complications, particularly pneumonia, whereas urinary tract infections were similarly distributed between groups. Finally, within the GA subgroup, delayed extubation was associated with worse neurological and functional outcomes, higher mortality, a substantially higher rate of pneumonia, and more frequent sICH. Taken together, these findings suggest that anesthetic strategy, baseline stroke severity, reperfusion success, infectious complications, and postprocedural airway management are closely interrelated determinants of outcome after thrombectomy. Although multivariable adjustment and propensity score matching were performed, residual confounding cannot be excluded. Therefore, the present study does not establish a causal harmful effect of GA.

Our findings should be interpreted in the context of randomized evidence comparing GA with conscious or procedural sedation during mechanical thrombectomy. Randomized trials, including SIESTA, AnStroke, GOLIATH, CANVAS, GASS, AMETIS, and SEGA, generally reported more balanced anesthesia groups, especially according to stroke severity, and used standardized anesthetic and hemodynamic protocols. The results of those trials did not consistently demonstrate worse functional outcome or higher mortality among patients treated under GA [12,13,14,15,16,17,18]. Several trials reported comparable or even numerically higher rates of successful reperfusion in the GA group, suggesting that, under standardized protocols and careful hemodynamic management, GA can be delivered without a clear detrimental effect on procedural or functional outcomes as shown in Supplementary Table S6. In GOLIATH, general anesthesia did not increase infarct growth compared with conscious sedation and was associated with signals of favorable procedural and clinical outcomes under protocolized anesthetic management. Similarly, AnStroke and GASS did not establish a clear functional advantage of conscious sedation. More recent trials also support an individualized interpretation: AMETIS reported similar functional and periprocedural outcomes between procedural sedation and general anesthesia, CANVAS II did not show superiority of conscious sedation in posterior circulation stroke, and SEGA suggested improved functional and reperfusion outcomes with general anesthesia compared with moderate sedation.

The discrepancy between these randomized data and our real-world cohort is clinically informative. In randomized trials, treatment allocation reduces selection bias, whereas in routine practice the choice of anesthesia is driven by clinical status and procedural considerations.

To further contextualize our findings, we performed an exploratory trial-level pooled comparison of randomized studies reporting binary outcomes after thrombectomy under GA versus conscious or procedural sedation (Supplementary Figure S4). Because outcome definitions and the reporting of complications varied across trials, pooled comparisons were performed on an exploratory basis and limited to endpoints with available trial-level data. Across randomized trials, GA was not associated with lower functional independence at 3 months (pooled OR 1.27, 95% CI 0.95–1.70) or higher 3-month mortality (pooled OR 0.91, 95% CI 0.66–1.26) (Supplementary Figure S4). By contrast, our real-world cohort showed substantially worse functional outcomes and higher mortality among patients treated under GA. This discrepancy supports the likely role of confounding by indication in routine clinical practice, where GA is more frequently selected for patients with more severe neurological deficits, impaired consciousness, airway compromise, posterior circulation strokes, or anticipated procedural complexity.

The persistence of an association between GA and worse 90-day outcomes after adjustment should therefore be interpreted cautiously. Although our models accounted for major prognostic factors, including age, baseline NIHSS, ASPECTS, vascular territory, treatment time, reperfusion success, number of thrombectomy passes, and blood pressure control, residual confounding cannot be fully excluded. Functional independence after MT is not determined solely by angiographic success or periprocedural management, but also by rehabilitation intensity and recovery trajectories after discharge. Moreover, unmeasured factors such as level of consciousness, aspiration before the procedure, agitation, hemodynamic instability including blood pressure variability, especially blood pressure drops during anesthesia, and peri-intubation complications may have influenced both the decision to intubate and subsequent outcomes. Thus, the observed association between GA and worse functional outcome or mortality should be interpreted as reflecting, at least in part, baseline stroke severity, clinical selection, procedural complexity, periprocedural management, and subsequent recovery, rather than as definitive evidence that GA itself is causally harmful [36].

An important finding of the present study is the higher rate of infectious complications, particularly pneumonia, among patients treated under GA. After multivariable adjustment, the association between GA and infectious outcomes showed only a non-significant trend, whereas no association was observed for urinary tract infection. This pattern suggests that the increased infectious burden in the GA group may be driven primarily by respiratory complications rather than by a generalized increase in nosocomial infections. Similar signals of increased pneumonia or pulmonary complications with GA have been reported in some randomized thrombectomy studies, although findings have not been consistent across trials [12,17,22]. The relationship between GA and pneumonia is biologically plausible, as endotracheal intubation, mechanical ventilation, impaired cough reflex, deeper sedation, secretion retention, and microaspiration may all contribute to pulmonary infection in the acute post-stroke period [19,20,37].

Pulmonary infections have been inconsistently reported across randomized trials, and definitions vary between studies. In our exploratory pooled comparison, randomized data showed a non-significant trend toward increased pulmonary infections with GA (pooled OR 1.74, 95% CI 0.85–3.56), whereas our real-world cohort demonstrated a clear association between GA and pneumonia (Supplementary Figure S4). This suggests that postprocedural respiratory complications may be an important and incompletely captured determinant of outcome after thrombectomy under GA, but the interpretation of pneumonia in our cohort requires caution. Although pneumonia was substantially more frequent among patients treated under GA in the unadjusted analysis, the association was attenuated after adjustment and did not reach statistical significance. This suggests that the higher pneumonia rate observed in the GA group may be partly explained by baseline stroke severity, impaired consciousness, aspiration risk, vascular territory, reperfusion-related factors, symptomatic intracranial hemorrhage, and the need for prolonged neurointensive care. Therefore, pneumonia should not be interpreted as an independently proven causal mediator between GA and poor outcome in this study. Rather, it may represent both a clinically relevant complication and a marker of more severe neurological injury or medical instability. In addition, our interval-specific mortality analysis suggested that the excess mortality associated with GA was most pronounced during the first 30 days after stroke, consistent with the broader concept that early post-stroke complications, particularly pneumonia, are important determinants of short-term outcomes [2,19,38].

The timing of extubation after thrombectomy under GA represents another clinically relevant aspect of postprocedural care. Earlier observational studies suggested that prolonged ventilation after thrombectomy may be associated with pneumonia, unfavorable functional outcome, and mortality. Nikoubashman et al. reported that prolonged ventilation was associated with pneumonia, poor outcome, and death, with ventilation beyond 24 h identifying patients at particularly high risk [39]. Similarly, Fandler-Höfler et al. found that shorter ventilation time correlated with better 3-month outcome, and, among patients extubated within 24 h, earlier extubation was associated with more favorable functional recovery [40].

Our findings are consistent with these observational data. Among patients treated under GA, delayed extubation beyond 6 h was associated with higher admission NIHSS scores, lower baseline ASPECTS, less frequent successful reperfusion, substantially higher rates of pneumonia, and worse neurological and functional outcomes during follow-up. These results suggest that prolonged intubation may identify a particularly vulnerable subgroup of patients after MT. Prolonged ventilation may contribute to pulmonary complications through impaired airway clearance, secretion retention, microaspiration, and ventilator-associated mechanisms. However, it may also reflect impaired consciousness, insufficient airway protection, respiratory instability, or neurological deterioration.

Recent randomized evidence further supports a cautious interpretation. The EDESTROKE randomized clinical trial compared early extubation (<6 h) with delayed extubation (6–12 h) after successful thrombectomy under GA and found no improvement in 90-day functional independence with early extubation [41]. Importantly, this trial included patients after successful reperfusion and compared relatively short ventilation intervals, which may partly explain the neutral findings. In contrast, our real-world cohort included a broader spectrum of patients, including those with more severe strokes and lower rates of successful reperfusion in the delayed extubation group. Therefore, our data should not be interpreted as proof that delayed extubation itself causes worse outcomes, but rather as evidence that prolonged postprocedural ventilation is closely linked to stroke severity, pulmonary complications, or hemorrhagic transformation, and adverse prognosis in routine practice. Accordingly, the extubation analysis should be considered exploratory and hypothesis-generating rather than causal.

From a clinical perspective, our findings support an individualized approach to anesthetic management during MT rather than a uniform preference for either GA or CS. This principle is consistent with other areas of neurological an neurosurgical anesthesia, such as awake craniotomy, where the choice of anesthetic technique is guided by the careful assessment of procedural requirements, patient cooperation, neurological status, and airway safety. In their systematic review and meta-analysis comparing the asleep–awake–asleep technique with monitored anesthesia care during awake craniotomy, Natalini et al. emphasized that patient selection and the tailoring of anesthetic approaches are central to achieving safe periprocedural management. A similar concept may apply to MT: when both GA and CS are clinically feasible, patient-specific factors should guide risk stratification and anesthetic planning rather than the routine use of one strategy in all patients.

In particular, elderly patients and those requiring admission to a neurointensive care unit may be more vulnerable to complications related to prolonged ventilation, impaired airway reflexes, aspiration, and pulmonary infection. Therefore, the duration of mechanical ventilation and the feasibility of safe early extubation should be considered clinically relevant components of the periprocedural care pathway. However, delayed extubation should not be interpreted solely as a modifiable exposure, because it may also reflect greater neurological injury, hemorrhagic complications, impaired consciousness, or respiratory instability [42].

### Limitations of the Study

Several limitations of this study should be acknowledged. First, this was a retrospective, single-center observational study, and therefore causal relationships between anesthetic strategy and clinical outcomes cannot be established. The choice of GA or CS was not randomized but was made according to the clinical condition of the patient and procedural considerations. As a result, confounding by indication is an important limitation [43]. Patients treated under GA presented with more severe strokes at baseline, including higher NIHSS scores and a greater proportion of posterior circulation strokes, suggesting that GA was preferentially used in clinically more unstable or severely affected patients. These same factors are also strong predictors of poor outcome after mechanical thrombectomy. Although multivariable adjustment and propensity score matching sensitivity analysis were performed, residual confounding cannot be excluded, particularly because important clinical variables such as level of consciousness, airway protection, aspiration before treatment, agitation, respiratory instability, and anesthetic depth were not fully captured in the retrospective dataset.

Second, although propensity score analysis and multivariable adjustment were performed for key prognostic variables, including age, baseline NIHSS and ASPECTS on admission, vascular territory, bridging rtPA, onset to TICI time, number of passes, successful reperfusion, blood pressure management and sICH occurrence, residual confounding cannot be excluded. Important factors that may have influenced both anesthesia choice and outcomes were not systematically available, including level of consciousness before treatment, agitation, vomiting, aspiration before intubation, airway protection status, intraprocedural blood pressure variability, oxygenation, ventilation parameters, used anesthetic agents, and depth of sedation or anesthesia.

Third, anesthesia and airway management were not protocolized. The decision to intubate, the timing of extubation, and postprocedural ventilatory management were based on clinical judgment rather than a standardized study protocol. Therefore, the observed association between delayed extubation and worse outcomes should be interpreted cautiously. Prolonged intubation may represent a marker of more severe neurological injury including sICH, impaired consciousness, or respiratory instability, rather than a purely modifiable exposure. Although our subgroup analysis among patients treated under GA provides clinically relevant information, it remains exploratory and is vulnerable to residual confounding.

Fourth, the analysis of infectious complications has inherent limitations. Pneumonia, urinary tract infection, and overall infectious complications were identified from clinical records, and standardized adjudication or microbiological confirmation was not available in all cases. This may have resulted in misclassification, particularly for pneumonia, which can be difficult to distinguish from aspiration pneumonitis, atelectasis, or other respiratory complications in severely affected stroke patients. In addition, because infections were recorded only during hospitalization, differences in length the of stay or intensity of monitoring between groups may have influenced detection rates [36,44].

Fifth, the study was limited by sample size, particularly in subgroup analyses and multivariable models. Although the overall cohort included 257 patients, the GA subgroup and the early versus delayed extubation comparison included smaller numbers of patients. This limits statistical power and increases the risk of unstable estimates, especially for less frequent outcomes such as urinary tract infection, specific antithrombotic treatments, or long-term mortality intervals. The subgroup analyses should therefore be considered hypothesis-generating.

Sixth, the multivariable models included a limited number of outcome events relative to the number of covariates, with events-per-variable ratios below the traditional threshold of 10. Therefore, some adjusted estimates, particularly those from exploratory models including symptomatic intracranial hemorrhage, may be imprecise and potentially unstable.

Seventh, infectious complications, including pneumonia, were identified retrospectively from clinical records and were not adjudicated by an independent blinded committee. As a result, underreporting, misclassification, or variability in diagnostic thresholds cannot be excluded.

Finally, although functional outcomes and mortality were assessed up to 12 months, follow-up data were obtained in the context of routine clinical care rather than a blinded trial protocol. The assessment of mRS may therefore be subject to measurement bias [45]. In addition, multiple outcomes and exploratory analyses were evaluated, increasing the possibility of type I error. For these reasons, our findings—particularly those concerning infectious complications and extubation timing—should be interpreted as exploratory and require confirmation in prospective studies with standardized anesthesia, ventilation, and infection-monitoring protocols.

## 5. Conclusions

In patients undergoing MT for AIS, anesthetic strategy appears to be closely linked to baseline stroke severity, postprocedural respiratory complications, and long-term outcome. Our findings suggest that the choice between GA and CS should not be considered in isolation, but rather as part of a broader periprocedural care pathway that includes careful patient selection, successful reperfusion, prevention of pulmonary complications, and timely extubation when feasible. Therefore, our findings should be interpreted as real-world associations affected by patient selection and residual confounding, rather than as evidence of the superiority of CS or causal harm from GA. These results support further prospective studies evaluating standardized anesthesia and airway-management protocols in endovascular stroke therapy.

## Figures and Tables

**Table 1:** Baseline and procedural characteristics of the study population.

Variable	Overall (*n* = 257)	Conscious Sedation (*n* = 155)	General Anesthesia (*n* = 102)	*p* Value
**Demographics and stroke characteristics**				
Age, years	72 (64–82)	72 (63–82)	72 (65–81)	0.90
Female sex	141/257 (54.9%)	83/155 (53.5%)	58/102 (56.9%)	0.60
Pre-stroke mRS	0 (0–0)	0 (0–0)	0 (0–0)	0.24
NIHSS on admission	16 (11–19)	14 (8–18)	17 (14–21)	<0.001
ASPECTS on admission	10 (8–10)	10 (8–10)	9.5 (8–10)	0.49
Anterior circulation	230/257 (89.5%)	145/155 (93.5%)	85/102 (83.3%)	0.009
Posterior circulation	27/257 (10.5%)	10/155 (6.5%)	17/102 (16.7%)	0.009
Intravenous thrombolysis (rtPA)	136/257 (52.9%)	81/155 (52.3%)	55/102 (53.9%)	0.79
**Comorbidities**				
Atrial fibrillation	125/257 (48.6%)	75/155 (48.4%)	50/102 (49.0%)	0.94
Hypertension	216/257 (84.0%)	130/155 (83.9%)	86/102 (84.3%)	0.92
Diabetes mellitus	74/257 (28.8%)	41/155 (26.5%)	33/102 (32.4%)	0.31
Current smoking	58/257 (22.6%)	34/155 (21.9%)	24/102 (23.5%)	0.77
Coronary artery disease	50/257 (19.5%)	32/155 (20.6%)	18/102 (17.6%)	0.55
Hyperlipidemia	98/257 (38.1%)	63/155 (40.6%)	35/102 (34.3%)	0.31
Previous TIA or stroke	34/257 (13.2%)	21/155 (13.5%)	13/102 (12.7%)	0.85
**Pre-stroke antiplatelet and anticoagulant therapy**				
Antiplatelet therapy (any)	47/257 (18.3%)	30/155 (19.4%)	17/102 (16.7%)	0.585
Aspirin	34/257 (13.2%)	21/155 (13.5%)	13/102 (12.7%)	0.852
Clopidogrel	2/257 (0.8%)	1/155 (0.6%)	1/102 (1.0%)	1.000
Other antiplatelet agent	3/257 (1.2%)	2/155 (1.3%)	1/102 (1.0%)	1.000
Dual antiplatelet therapy (DAPT)	8/257 (3.1%)	6/155 (3.9%)	2/102 (2.0%)	0.484
Anticoagulation (any)	56/257 (21.8%)	34/155 (21.9%)	22/102 (21.6%)	0.94
NOAC	27/257 (10.5%)	16/155 (10.3%)	11/102 (10.8%)	0.91
VKA	21/257 (8.2%)	11/155 (7.1%)	10/102 (9.8%)	0.44
LMWH	8/257 (3.1%)	7/155 (4.5%)	1/102 (1.0%)	0.15
Pre-stroke statin therapy	71/257 (27.6%)	42/155 (27.1%)	29/102 (28.4%)	0.81
**Procedural characteristics**				
OTG, hours	3.77 (2.53–5.27)	3.98 (2.56–5.29)	3.65 (2.50–5.07)	0.51
OTTICI, hours	4.61 (3.29–6.13)	4.57 (3.20–6.16)	4.67 (3.39–6.08)	0.75
Number of thrombectomy passes	2 (1–3)	2 (1–3)	2 (2–3)	0.002
Successful reperfusion (TICI 2b–3)	217/257 (84.4%)	135/155 (87.1%)	82/102 (80.4%)	0.15
SBP at beginning of anesthesia (mmHg)	150.0 (133.5–171.0)	148.5 (133–166)	151 (134.5–177.5)	0.31
DBP at beginning of anesthesia (mmHg)	83.0 (74.5–97.0)	81 (74–93.25)	86 (76.5–100)	0.16
SBP at end of anesthesia (mmHg)	140.0 (129.0–160.0)	140 (130–157)	140 (125–174)	0.70
DBP at end of anesthesia (mmHg)	80.0 (70.0–90.0)	80 (70–90)	80 (70–95)	0.66

Continuous and ordinal variables were presented as median (IQR) and compared using the Mann–Whitney U test. Binary variables were presented as *n*/N (%) and compared using the χ^2^ test. *p* values refer to comparisons between CS and GA. NIHSS, National Institutes of Health Stroke Scale; mRS, modified Rankin Scale; ASPECTS, Alberta Stroke Program Early CT Score; NOAC, non-vitamin K antagonist oral anticoagulant; VKA, vitamin K antagonist; LMWH, low-molecular-weight heparin; OTG, onset-to-groin; OTTICI, onset-to-TICI; SBP, systolic blood pressure; and DBP, diastolic blood pressure.

**Table 2:** Clinical outcomes according to anesthesia strategy.

Outcome	Overall (*n* = 257)	Conscious Sedation (*n* = 155)	General Anesthesia (*n* = 102)	*p* Value
NIHSS at 24 h, median (IQR)	11 (6–18)	8 (4–14)	18 (12–22)	<0.001
ASPECTS at 24 h, median (IQR)	7 (5–8)	7 (5–8)	6 (3–7)	0.005
Any ICH, *n* (%)	96/257 (37.3%)	53/155 (34.2%)	43/102 (42.2%)	0.197
sICH, *n* (%)	29/257 (11.3%)	11/155 (7.1%)	18/102 (17.6%)	0.009
aICH, *n* (%)	67/257 (26.1%)	42/155 (27.1%)	25/102 (24.5%)	0.644
NIHSS at discharge, median (IQR)	6 (2–13)	4 (1–9)	9 (4–16)	<0.001
mRS at discharge, median (IQR)	4 (3–6)	4 (2–5)	5 (4–6)	<0.001
mRS at 30 days, median (IQR)	4 (2–6)	3 (1–5)	5 (3–6)	<0.001
mRS at 90 days, median (IQR)	4 (2–6)	3 (1–5)	5 (3–6)	<0.001
mRS at 12 months, median (IQR)	4 (2–6)	3 (1–6)	5 (3–6)	<0.001
Functional independence at 90 days (mRS 0–2), *n* (%)	93/257 (36.2%)	71/155 (45.8%)	22/102 (21.6%)	<0.001
Functional independence at 12 months (mRS 0–2), *n* (%)	99/257 (38.5%)	75/155 (48.4%)	24/102 (23.5%)	<0.001
In-hospital mortality, *n* (%)	48/257 (18.7%)	20/155 (12.9%)	28/102 (27.5%)	0.003
30-day mortality, *n* (%)	51/257 (19.8%)	22/155 (14.2%)	29/102 (28.4%)	0.005
90-day mortality, *n* (%)	78/257 (30.4%)	35/155 (22.6%)	43/102 (42.2%)	<0.001
12-month mortality, *n* (%)	100/257 (38.9%)	50/155 (32.3%)	50/102 (49.0%)	0.007

Ordinal variables were presented as median (IQR) and compared using the Mann–Whitney U test. Binary variables were presented as *n*/N (%) and compared using the χ^2^ test. *p* values refer to comparisons between CS and GA. NIHSS, National Institutes of Health Stroke Scale; mRS, modified Rankin Scale; ASPECTS, Alberta Stroke Program Early CT Score; ICH, Intracranial hemorrhage, sICH symptomatic intracranial hemorrhage, aICH asymptomatic intracranial hemorrhage.

**Table 3:** Interval-specific mortality according to anesthetic strategy.

Time Interval	Overall	Conscious Sedation	General Anesthesia	*p* Value
0–30 days	51/257 (19.8%)	22/155 (14.2%)	29/102 (28.4%)	0.005
31–90 days	27/206 (13.1%)	13/133 (9.8%)	14/73 (19.2%)	0.056
91 days–12 months	22/179 (12.3%)	15/120 (12.5%)	7/59 (11.9%)	0.903

Variables are presented as *n*/N (%) and compared using the χ^2^ test. *p* values refer to comparisons between CS and GA.

**Table 4:** Infectious complications according to anesthesia strategy.

Outcome	Overall (*n* = 257)	Conscious Sedation (*n* = 155)	General Anesthesia (*n* = 102)	*p* Value
Any infectious complication	94/257 (36.6%)	44/155 (28.4%)	50/102 (49.0%)	0.001
Pneumonia	91/257 (35.4%)	41/155 (26.5%)	50/102 (49.0%)	<0.001
Urinary tract infection	46/257 (17.9%)	28/155 (18.1%)	18/102 (17.6%)	0.932

Variables are presented as *n*/N (%) and compared using the χ^2^ test. *p* values refer to comparisons between CS and GA.

## Data Availability

The data presented in this study are available from the corresponding author upon reasonable request, subject to institutional and ethical restrictions.

## Supplementary Materials

The following supporting information can be downloaded at https://www.mdpi.com/article/10.3390/jcm15134993/s1. Supplementary Table S1: Occluded vessels according to anesthetic strategy; Supplementary Table S2: Model stability diagnostics for the main multivariable logistic regression model; Supplementary Table S3: Covariate balance before and after propensity score matching; Supplementary Table S4: Outcomes after propensity score matching; Supplementary Table S5: Exploratory subgroup analysis among patients treated under general anesthesia: early (<6 h) vs delayed (>6 h) extubation; Supplementary Table S6: Randomized studies investigating type of anesthesia; Supplementary Figure S1: Additional exploratory adjusted analysis for mortality at 90 days; Supplementary Figure S2: Additional exploratory adjusted analysis functional independence at 90 days. Supplementary Figure S3: Love plot before and after propensity score matching. Cohort balance before and after propensity score matching; and Supplementary Figure S4: Exploratory pooled comparison of randomized trials evaluating general anesthesia versus conscious/procedural sedation during mechanical thrombectomy by functional independence at 3 months (mRS = 0–2), mortality at 3 months and pulmonary infections.

## Abbreviations

The following abbreviations are used in this manuscript:

Abbreviation	Definition
ACA	Anterior Cerebral Artery
AHA/ASA	American Heart Association/American Stroke Association
aICH	Asymptomatic Intracranial Hemorrhage
AIS	Acute Ischemic Stroke
AMETIS	Anesthesia Management in Endovascular Therapy for Ischemic Stroke
AnStroke	Anesthesia During Stroke
ASPECTS	Alberta Stroke Program Early CT Score
BA	Basilar Artery
BP	Blood Pressure
CANVAS	Choice of ANesthesia for EndoVAScular Treatment of Acute Ischemic Stroke
CDC/NHSN	Centers for Disease Control and Prevention/National Healthcare Safety Network
CI	Confidence Interval
CS	Conscious Sedation
CT	Computed Tomography
DAPT	Dual Antiplatelet Therapy
DBP	Diastolic Blood Pressure
EDESTROKE	Early vs. Delayed Extubation After Endovascular Treatment for Acute Ischemic Stroke
ESO	European Stroke Organisation
ESO-ESMINT	European Stroke Organisation–European Society of Minimally Invasive Neurological Therapy
GA	General Anesthesia
GASS	General Anesthesia vs. Sedation for Stroke
GOLIATH	General or Local Anesthesia in Intra-Arterial Therapy
ICA	Internal Carotid Artery
ICH	Intracranial Hemorrhage
IQR	Interquartile Range
LMWH	Low-Molecular-Weight Heparin
LVO	Large-Vessel Occlusion
MCA	Middle Cerebral Artery
mRS	Modified Rankin Scale
MT	Mechanical Thrombectomy
NA	Not Applicable
NIHSS	National Institutes of Health Stroke Scale
NOAC	Non-Vitamin K Antagonist Oral Anticoagulant
OR	Odds Ratio
OTG	Onset-To-Groin Time
OTTICI	Onset-To-Tici Time
PCA	Posterior Cerebral Artery
rtPA	Recombinant Tissue Plasminogen Activator
SBP	Systolic Blood Pressure
SEGA	Sedation Versus General Anesthesia for Endovascular Therapy in Acute Ischemic Stroke
sICH	Symptomatic Intracranial Hemorrhage
SIESTA	Sedation vs. Intubation for Endovascular Stroke Treatment
STROBE	Strengthening the Reporting of Observational Studies in Epidemiology
TIA	Transient Ischemic Attack
TICI	Thrombolysis in Cerebral Infarction
VA	Vertebral Artery
VKA	Vitamin K Antagonist
