# Supplementary material for: Anesthetic Strategy, Functional Outcomes, and Infectious Complications After Mechanical Thrombectomy for Acute Ischemic Stroke
Source: J Clin Med. 2026 Jun 26;15(13):4993. doi: 10.3390/jcm15134993 (PMC13362634; doi:10.3390/jcm15134993)
Supplement: Supplementary file 1 [file jcm-15-04993-s001.zip › Supplementary Figure S4 Exploratory pooled comparison of randomized trials evaluating.pdf]

Supplementary Figure S4: Exploratory pooled comparison of randomized trials evaluating general anesthesia versus conscious/procedural sedation during mechanical thrombectomy by functional independence at 3 months (mRS= 0-2), mortality at 3 months and pulmonary infections.

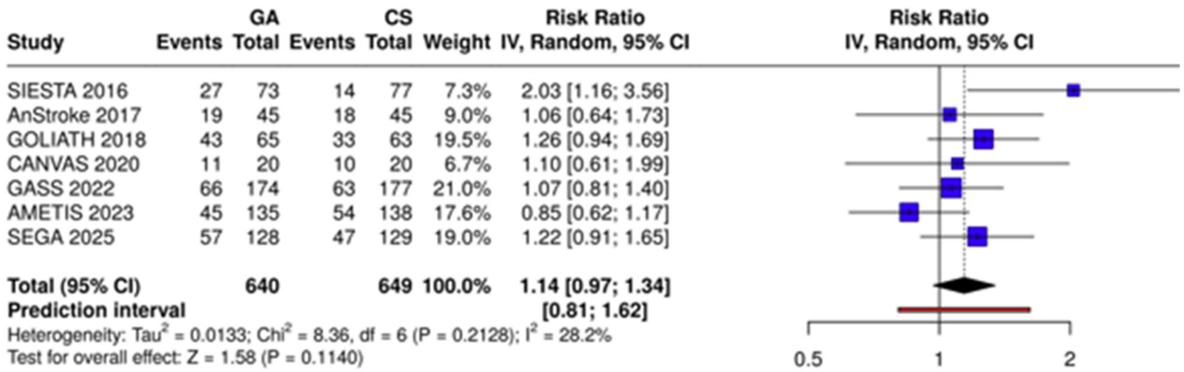

Study	GA Events	GA Total	CS Events	CS Total	Weight	Risk Ratio IV, Random, 95% CI
SIESTA 2016	27	73	14	77	7.3%	2.03 [1.16; 3.56]
AnStroke 2017	19	45	18	45	9.0%	1.06 [0.64; 1.73]
GOLIATH 2018	43	65	33	63	19.5%	1.26 [0.94; 1.69]
CANVAS 2020	11	20	10	20	6.7%	1.10 [0.61; 1.99]
GASS 2022	66	174	63	177	21.0%	1.07 [0.81; 1.40]
AMETIS 2023	45	135	54	138	17.6%	0.85 [0.62; 1.17]
SEGA 2025	57	128	47	129	19.0%	1.22 [0.91; 1.65]
**Total (95% CI)**		**640**		**649**	**100.0%**	**1.14 [0.97; 1.34]**
**Prediction interval**						**[0.81; 1.62]**

Heterogeneity: $Tau^2 = 0.0133$; $Chi^2 = 8.36$, df = 6 (P = 0.2128); $I^2 = 28.2\%$
Test for overall effect: Z = 1.58 (P = 0.1140)

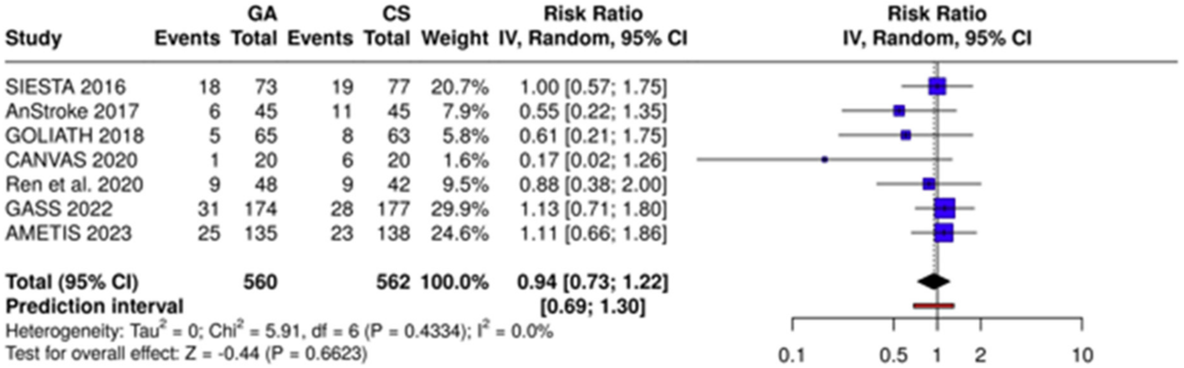

Study	GA Events	GA Total	CS Events	CS Total	Weight	Risk Ratio IV, Random, 95% CI
SIESTA 2016	18	73	19	77	20.7%	1.00 [0.57; 1.75]
AnStroke 2017	6	45	11	45	7.9%	0.55 [0.22; 1.35]
GOLIATH 2018	5	65	8	63	5.8%	0.61 [0.21; 1.75]
CANVAS 2020	1	20	6	20	1.6%	0.17 [0.02; 1.26]
Ren et al. 2020	9	48	9	42	9.5%	0.88 [0.38; 2.00]
GASS 2022	31	174	28	177	29.9%	1.13 [0.71; 1.80]
AMETIS 2023	25	135	23	138	24.6%	1.11 [0.66; 1.86]
**Total (95% CI)**		**560**		**562**	**100.0%**	**0.94 [0.73; 1.22]**
**Prediction interval**						**[0.69; 1.30]**

Heterogeneity: $Tau^2 = 0$; $Chi^2 = 5.91$, df = 6 (P = 0.4334); $I^2 = 0.0\%$
Test for overall effect: Z = -0.44 (P = 0.6623)

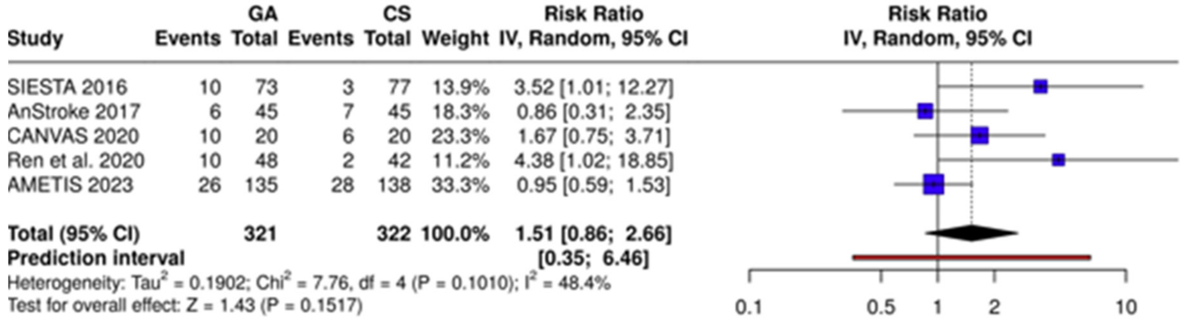

Study	GA Events	GA Total	CS Events	CS Total	Weight	Risk Ratio IV, Random, 95% CI
SIESTA 2016	10	73	3	77	13.9%	3.52 [1.01; 12.27]
AnStroke 2017	6	45	7	45	18.3%	0.86 [0.31; 2.35]
CANVAS 2020	10	20	6	20	23.3%	1.67 [0.75; 3.71]
Ren et al. 2020	10	48	2	42	11.2%	4.38 [1.02; 18.85]
AMETIS 2023	26	135	28	138	33.3%	0.95 [0.59; 1.53]
**Total (95% CI)**		**321**		**322**	**100.0%**	**1.51 [0.86; 2.66]**
**Prediction interval**						**[0.35; 6.46]**

Heterogeneity: $Tau^2 = 0.1902$; $Chi^2 = 7.76$, df = 4 (P = 0.1010); $I^2 = 48.4\%$
Test for overall effect: Z = 1.43 (P = 0.1517)

An exploratory trial-level pooled comparison of randomized studies was performed with metaanalysisonline.com for binary outcomes with available event counts. Odds ratios were calculated for general anesthesia versus conscious/procedural sedation. Randomized trials were pooled using a random-effects model. The present observational cohort is shown separately as a real-world comparator and was not included in the pooled randomized estimate. For functional independence, OR >1 favors general anesthesia; for mortality and pulmonary infections, OR >1 indicates higher risk with general anesthesia.
