# Supplementary material for: Anesthetic Strategy, Functional Outcomes, and Infectious Complications After Mechanical Thrombectomy for Acute Ischemic Stroke
Source: J Clin Med. 2026 Jun 26;15(13):4993. doi: 10.3390/jcm15134993 (PMC13362634; doi:10.3390/jcm15134993)
Supplement: Supplementary file 1 [file jcm-15-04993-s001.zip › Supplementary Table S1. Occluded vessels according to anesthetic strategy.pdf]

Supplementary Table S1. Occluded vessels according to anesthetic strategy

Occluded vessel	Overall (n = 257)	Conscious sedation (n = 155)	General anesthesia (n = 102)
MCA M1	109/257 (42.4%)	73/155 (47.1%)	36/102 (35.3%)
MCA M2-M3	59/257 (23.0%)	40/155 (25.8%)	19/102 (18.6%)
T-occlusion	45/257 (17.5%)	25/155 (16.1%)	20/102 (19.6%)
ACA	2/257 (0.8%)	2/155 (1.3%)	0/102 (0.0%)
PCA	8/257 (3.1%)	7/155 (4.5%)	1/102 (1.0%)
BA	18/257 (7.0%)	3/155 (1.9%)	15/102 (14.7%)
ICA	15/257 (5.8%)	5/155 (3.2%)	10/102 (9.8%)
VA	1/257 (0.4%)	0/155 (0.0%)	1/102 (1.0%)

Values are presented as n/N (%).MCA, middle cerebral artery; ACA, anterior cerebral artery; PCA, posterior cerebral artery; BA, basilar artery; ICA, internal carotid artery; VA, vertebral artery.
