# Supplementary material for: Anesthetic Strategy, Functional Outcomes, and Infectious Complications After Mechanical Thrombectomy for Acute Ischemic Stroke
Source: J Clin Med. 2026 Jun 26;15(13):4993. doi: 10.3390/jcm15134993 (PMC13362634; doi:10.3390/jcm15134993)
Supplement: Supplementary file 1 [file jcm-15-04993-s001.zip › Supplementary Table S2. Model stability diagnostics for the main multivariable logistic regression models.pdf]

Supplementary Table S2. Model stability diagnostics for the main multivariable logistic regression models

Outcome model	Complete-case N	Events, n	Non-events, n	Covariates, n	EPV	AUC	AUC 95% CI	Hosmer-Lemeshow p-value	VIF range
90-day mortality	187	55	132	11	5.0	0.852	0.783-0.922	0.251	1.12-1.34
90-day functional independence	189	69	120	11	6.3	0.832	0.759-0.905	0.940	1.10-1.37
Pneumonia	185	61	124	11	5.5	0.802	0.718-0.885	0.276	1.11-1.31
Any infectious complication	185	64	121	11	5.8	0.773	0.682-0.865	0.999	1.09-1.35

EPV, events-per-variable ratio; AUC, area under the receiver operating characteristic curve; VIF, variance inflation factor. Events were defined as the number of patients experiencing the modeled outcome. EPV was calculated as the number of events divided by the number of covariates included in the model. Calibration was assessed using the Hosmer-Lemeshow goodness-of-fit test. Multicollinearity was assessed using variance inflation factors.
