# Supplementary material for: Anesthetic Strategy, Functional Outcomes, and Infectious Complications After Mechanical Thrombectomy for Acute Ischemic Stroke
Source: J Clin Med. 2026 Jun 26;15(13):4993. doi: 10.3390/jcm15134993 (PMC13362634; doi:10.3390/jcm15134993)
Supplement: Supplementary file 1 [file jcm-15-04993-s001.zip › Supplementary Table S3. Covariate balance before and after propensity score matching..pdf]

Supplementary Table S3. Covariate balance before and after propensity score matching.

Variable	GA before matching (mean/proportion)	CS before matching (mean/proportion)	SMD before matching	GA after matching (mean/proportion)	CS after matching (mean/proportion)	SMD after matching
Propensity score	0.511	0.336	0.900	0.511	0.431	0.429
Age, per decade	7.086	6.969	0.082	7.086	7.021	0.046
Female sex	0.566	0.595	-0.058	0.566	0.529	0.076
Pre-stroke mRS	0.301	0.306	-0.006	0.301	0.171	0.203
Baseline NIHSS	17.783	13.612	0.726	17.783	16.357	0.264
Posterior circulation	0.157	0.041	0.394	0.157	0.071	0.270
Intravenous thrombolysis	0.542	0.512	0.060	0.542	0.543	-0.001
Baseline ASPECTS	8.892	9.132	-0.185	8.892	9.100	-0.156
Atrial fibrillation	0.494	0.479	0.029	0.494	0.471	0.045
Hypertension	0.819	0.851	-0.086	0.819	0.857	-0.103
Diabetes mellitus	0.301	0.256	0.101	0.301	0.271	0.066
Current smoking	0.229	0.231	-0.006	0.229	0.271	-0.098
Coronary artery disease	0.181	0.174	0.019	0.181	0.129	0.145
Hyperlipidemia	0.349	0.364	-0.030	0.349	0.300	0.106
Previous TIA/stroke	0.145	0.165	-0.057	0.145	0.129	0.047

Values are presented as means or proportions. SMD, standardized mean difference; GA, general anesthesia; CS, conscious sedation; mRS, modified Rankin Scale; NIHSS, National Institutes of Health Stroke Scale; ASPECTS, Alberta Stroke Program Early CT Score; TIA, transient ischemic attack; rtPA, recombinant tissue plasminogen activator.
