# Supplementary material for: Anesthetic Strategy, Functional Outcomes, and Infectious Complications After Mechanical Thrombectomy for Acute Ischemic Stroke
Source: J Clin Med. 2026 Jun 26;15(13):4993. doi: 10.3390/jcm15134993 (PMC13362634; doi:10.3390/jcm15134993)
Supplement: Supplementary file 1 [file jcm-15-04993-s001.zip › Supplementary Table S4 Outcomes after propensity score matching..pdf]

Supplementary Table S4. Outcomes after propensity score matching.

Outcome	General anesthesia (n = 70)	Conscious sedation (n = 70)	McNemar p value
Functional independence at 90 days	15/70 (21.4%)	26/70 (37.1%)	0.052
90-day mortality	29/70 (41.4%)	16/70 (22.9%)	0.029
Pneumonia	33/70 (47.1%)	20/70 (28.6%)	0.053
Any infectious complication	30/70 (42.9%)	19/70 (27.1%)	0.108

Values are presented as n/N (%). Comparisons between matched pairs were performed using McNemar's test. GA, general anesthesia; CS, conscious sedation.
