# Supplementary material for: Anesthetic Strategy, Functional Outcomes, and Infectious Complications After Mechanical Thrombectomy for Acute Ischemic Stroke
Source: J Clin Med. 2026 Jun 26;15(13):4993. doi: 10.3390/jcm15134993 (PMC13362634; doi:10.3390/jcm15134993)
Supplement: Supplementary file 1 [file jcm-15-04993-s001.zip › Supplementary Table S5. Exploratory subgroup analysis among patients treated under general anesthesia.pdf]

Supplementary Table S5. Exploratory subgroup analysis among patients treated under general anesthesia: early (≤6 h) vs delayed (>6 h) extubation

Variable	Early extubation ≤6 h (n = 55)	Delayed extubation >6 h (n = 47)	P value
**Baseline characteristics**			
Female sex, n (%)	33/55 (60.0%)	25/47 (53.2%)	0.489
Age, decades	7.2 (6.5-8.1)	7.2 (6.3-8.1)	0.786
NIHSS on admission	16 (14-19)	19 (16-22)	0.017
ASPECTS on admission	10 (9-10)	8.5 (8-10)	0.006
Anterior circulation, n (%)	48/55 (87.3%)	37/47 (78.7%)	0.248
Posterior circulation, n (%)	7/55 (12.7%)	10/47 (21.3%)	0.248
**Procedural characteristics**			
Intravenous thrombolysis (rtPA), n (%)	34/55 (61.8%)	25/47 (53.2%)	0.379
OTG, hours	3.8 (2.4-5.3)	3.3 (2.6-5.0)	0.689
OTTICI, hours	4.8 (3.8-6.2)	4.5 (3.2-5.9)	0.435
Number of thrombectomy passes	2 (1-3)	2 (2-3)	0.466
Successful reperfusion (TICI 2b-3), n (%)	49/55 (89.1%)	33/47 (70.2%)	0.017
Any ICH, n (%)	16/55 (29.1%)	27/47 (57.4%)	0.004
sICH, n (%)	2/55 (3.6%)	16/47 (34.0%)	<0.001
aICH, n (%)	14/55 (25.5%)	11/47 (23.4%)	0.810
SBP, beginning of anesthesia (mmHg)	145.0 (131.3-170.0)	161.0 (142.0-179.0)	0.147
DBP, beginning of anesthesia (mmHg)	83.0 (74.3-100.0)	89.0 (79.5-100.5)	0.389
SBP, end of anesthesia (mmHg)	144.0 (127.0-178.8)	140.0 (122.0-160.0)	0.382
DBP, end of anesthesia (mmHg)	80.0 (70.0-100.0)	82.0 (70.0-90.5)	0.921
**Pre-stroke pharmacotherapy**			
Antiplatelet therapy (any), n (%)	7/55 (12.7%)	10/47 (21.3%)	0.248
Aspirin, n (%)	5/55 (9.1%)	8/47 (17.0%)	0.231
Clopidogrel, n (%)	0/55 (0.0%)	1/47 (2.1%)	0.461
Other antiplatelet agent, n (%)	1/55 (1.8%)	1/47 (2.1%)	1.000
Dual antiplatelet therapy (DAPT), n (%)	0/55 (0.0%)	0/47 (0.0%)	NA
Anticoagulation (any), n (%)	10/55 (18.2%)	8/47 (17.0%)	0.878
NOAC, n (%)	6/55 (10.9%)	5/47 (10.6%)	0.965
VKA, n (%)	5/55 (9.1%)	5/47 (10.6%)	1.000
LMWH, n (%)	0/55 (0.0%)	1/47 (2.1%)	0.461
Pre-stroke statin therapy, n (%)	15/55 (27.3%)	14/47 (29.8%)	0.779
**Clinical and functional outcomes**			
NIHSS at discharge	7 (3-13)	15 (6-20)	0.019
mRS at discharge	4 (2-5)	5 (5-6)	<0.001
mRS at 30 days	4 (2-5)	5 (5-6)	<0.001
mRS at 90 days	4 (2-5)	6 (4-6)	<0.001
mRS at 12 months	4 (2-6)	6 (4-6)	0.002
Functional independence at 90 days (mRS 0-2), n (%)	15/55 (27.3%)	7/47 (14.9%)	0.130
30-day mortality, n (%)	8/55 (14.5%)	21/47 (44.7%)	0.001
90-day mortality, n (%)	13/55 (23.6%)	30/47 (63.8%)	<0.001
12-month mortality, n (%)	18/55 (32.7%)	32/47 (68.1%)	<0.001
**Infectious complications**			
Any infectious complication, n (%)	19/55 (34.5%)	31/47 (66.0%)	0.002
Pneumonia, n (%)	17/55 (30.9%)	33/47 (70.2%)	<0.001
Urinary tract infection, n (%)	8/55 (14.5%)	10/47 (21.3%)	0.300

Continuous and ordinal variables were presented as median (IQR) and compared using the Mann–Whitney U test. Binary variables were presented as n/N (%) and compared using the χ^2 test. P values refer to comparisons between early and delayed extubation group. Analyses are restricted to patients treated under general anesthesia. NIHSS, National Institutes of Health Stroke Scale; ASPECTS, Alberta Stroke Program Early CT Score; OTG, onset-to-groin; OTTICI, onset-to-TICI; TICI, Thrombolysis in Cerebral Infarction; ICH, intracranial hemorrhage; sICH, symptomatic intracranial hemorrhage; aICH, asymptomatic intracranial hemorrhage; SBP, systolic blood pressure; DBP, diastolic blood pressure; NOAC, non-vitamin K antagonist oral anticoagulant; VKA, vitamin K antagonist; LMWH, low-molecular-weight heparin; mRS, modified Rankin Scale; NA, not applicable.
