# Supplementary material for: Anesthetic Strategy, Functional Outcomes, and Infectious Complications After Mechanical Thrombectomy for Acute Ischemic Stroke
Source: J Clin Med. 2026 Jun 26;15(13):4993. doi: 10.3390/jcm15134993 (PMC13362634; doi:10.3390/jcm15134993)
Supplement: Supplementary file 1 [file jcm-15-04993-s001.zip › Supplementary Table S6. Randomized studies investigating type of anesthesia..pdf]

Supplementary Table S6. Randomized studies investigating type of anesthesia.

Author, year	N of patients	Type of anesthesia (n)	NIHSS admission Median (IQR)	ASPECTS admission Median (IQR)	TICI 2b-3 n (%)	mRS 3 month Median (IQR)	mRS 0-2 3 months n(%)	Mortality 3 months n(%)	Pulmonary Infections n(%)
Dębiec et al.	257	GA: 102<br>CS: 155	GA: 17 (14–21)<br>CS: 14 (8–18)	GA: 9.5 (8–10)<br>CS: 10 (8–10)	GA: 82 (80.4%)<br>CS:135 (87.1%)	GA: 5 (3–6)<br>CS: 3 (1–5)	GA: 22 (21.6%)<br>CS: 71 (45.8%)	GA: 43 (42.2%)<br>CS: 35 (22.6%)	GA: 50 (49.0%)<br>CS:41 (26.5%)
SIESTA—Schönenberger et al.(2016)	150	GA: 73<br>CS: 77	GA: 17 (13-20)<br>CS: 17 (14-20)	GA: 8 (7-9)<br>CS: 8 (6.25-9)	GA:65 (89%)<br>CS: 62 (80.5%)	GA:4 (2- 5)<br>CS:4 (3- 5)	GA:27 (37.0%)<br>CS: 14 (18.2%)	GA: 18 (24.7%)<br>CS 19 (24.7%)	*GA:13,7%<br>CS: 3,9 %
AnStroke — Löwhagen Hendén et al. (2017)	90	GA: 45<br>CS: 45	GA: 20 (15.5–23)<br>CS:17 (14–20.5)	GA: 10 (8–10)<br>CS: 10 (9–10)	GA: 41 (91.1%)<br>CS: 40 (88.9%)	GA: 3 (1–4)<br>CS:3 (1–5.5)	GA: 19 (42.2%)<br>CS: 18 (40.0%)	GA: 6 (13.3%)<br>CS: 11 (24.4%)	GA: 6 (13.3 %)<br>CS: 7 (15.6%)
GOLIATH — Simonsen et al. (2018)	128	GA: 65<br>CS: 63	GA: 18 (13–21)<br>CS: 17 (15– 21)	n/a	GA: 50 (76.9%)<br>CS: 38 (60.3%)	GA:2 (1–3)<br>CS:2 (1–4)	GA: 43 (66.1%)<br>CS: 33 (52.4%)	GA: 5 (7.7%)<br>CS: 8 (12.7%)	n/a
CANVAS pilot — Sun et al. (2020)	40	GA: 20<br>CS: 20	GA: 14 (11–18)<br>CS: 13 (9–17)	n/a	GA: 19 (95%)<br>CS:13 (65%)	GA:2 (1- 4)<br>CS: 3 (1- 6)	GA:11 (55%)<br>CS: 10 (50%)	GA: 1 (5%)<br>CS: 6 (30%)	GA: 10 (50%)<br>CS: 6 (30%)
Ren et al. (2020)	90	GA: 48<br>CS: 42	GA: 14 (11–16)<br>CS: 14 (11–16)	GA:9 (8–10)<br>CS: 9 (8–10.3)	GA:42 (87.5%)<br>CS: 36 (85.7%)	GA: 2.5 (2–3)<br>CS:2.5 (2–3)	n/a	GA: 9 (18.8%)<br>CS: 9 (20.9%)	GA: 10 (20.8%)<br>CS: 2 (4.8%)
GASS — Maurice et al. (2022)	345	GA:174<br>CS: 177	**GA: 16 (SD ±6)<br>CS: 16 (SD ±5)	n/a	GA: 144 (85%)<br>CS: 131 (75%)	n/a	GA:66 (40%)<br>CS: 63 (36%)	GA: 31 (19%)<br>CS: 28 (16%)	n/a
AMETIS — Chabanne et al. (2023)	273	GA: 135<br>CS: 138	GA: 16 (11–20)<br>CS: 15 (11–20)	GA: 8 (7–9)<br>CS: 8 (7–9)	GA: 115(85.2%)<br>CS: 107 (77.6%)	GA: 3 (2–5)<br>CS: 3 (2–4)	GA:45 (33.3%)<br>CS: 54 (39.1%)	GA: 25 (18.5%)<br>CS: 23 (16.7%)	GA: 26 (19.3%)<br>CS: 28 (20.4%)
SEGA-Chen et al (2025)	257	GA: 128<br>CS: 129	GA: 15 (10-18)<br>CS: 16 (11-20)	GA: 8 (7-10)<br>CS: 9 (7-10)	GA: 118 (96,7%)<br>CS: 116 (95%)	GA: 3 (1-5)<br>CS: 3 (1-5)	GA: 57 (48%)<br>CS: 47 (39%)	n/a	n/a

Data extracted from published randomized trials; values reported as provided in the original manuscripts;* Authors provided only % value ;** Authors provided mean NIHSS on admission.
