# Supplementary figures and images for: Anesthetic Strategy, Functional Outcomes, and Infectious Complications After Mechanical Thrombectomy for Acute Ischemic Stroke
Source: J Clin Med. 2026 Jun 26;15(13):4993. doi: 10.3390/jcm15134993 (PMC13362634; doi:10.3390/jcm15134993)

Supplementary Figure S1: Additional adjusted analysis for mortality at 90 days

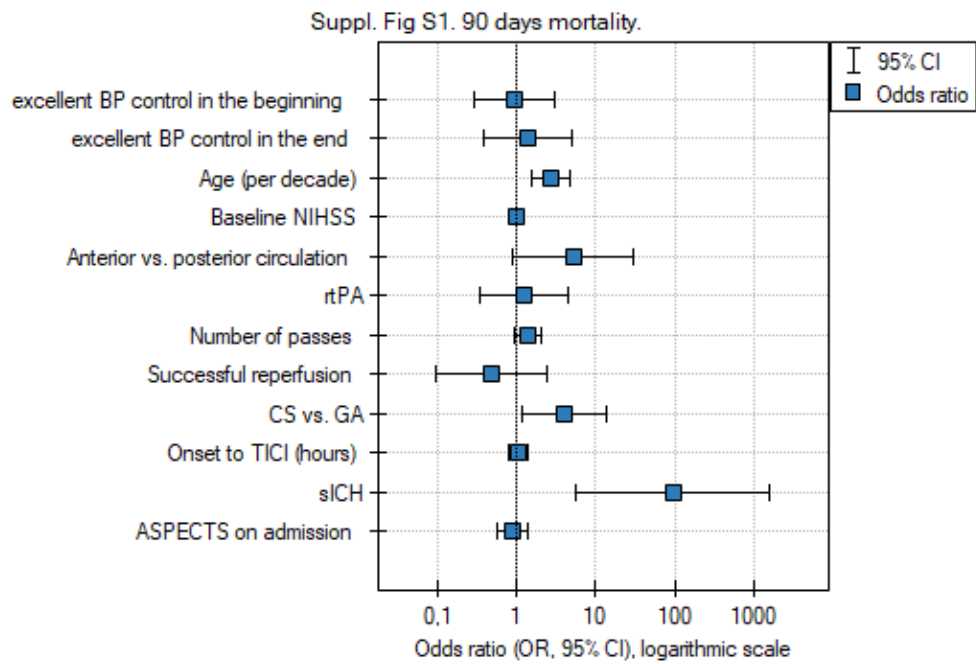

Supplement: Supplementary file 1 [file jcm-15-04993-s001.zip › Supplementary Figure S1 Additional adjusted analysis for mortality at 90 days.pdf]

Supplementary Figure S2: Additional adjusted analysis for functional independence at 90 days

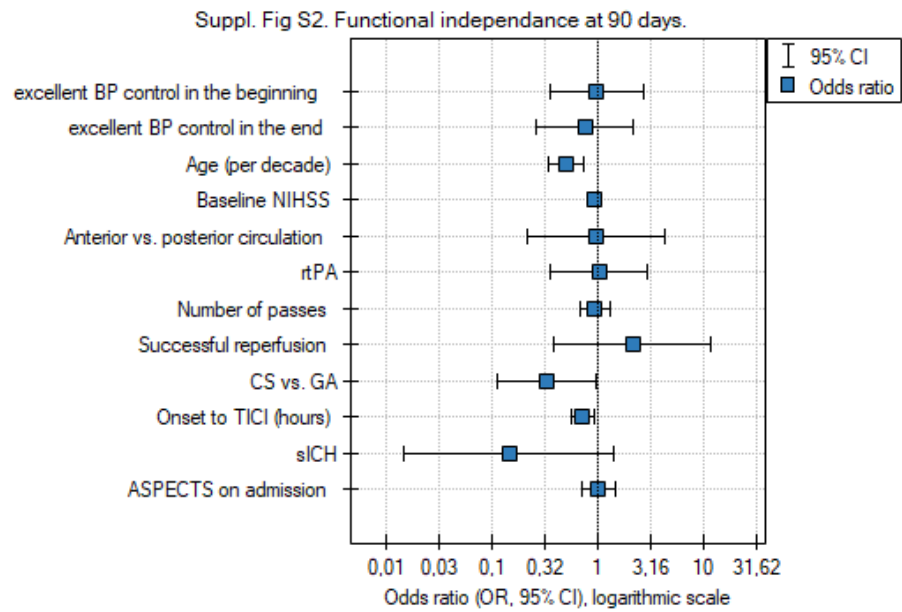

Supplement: Supplementary file 1 [file jcm-15-04993-s001.zip › Supplementary Figure S2 Additional adjusted analysis for functional independence at 90 days.pdf]

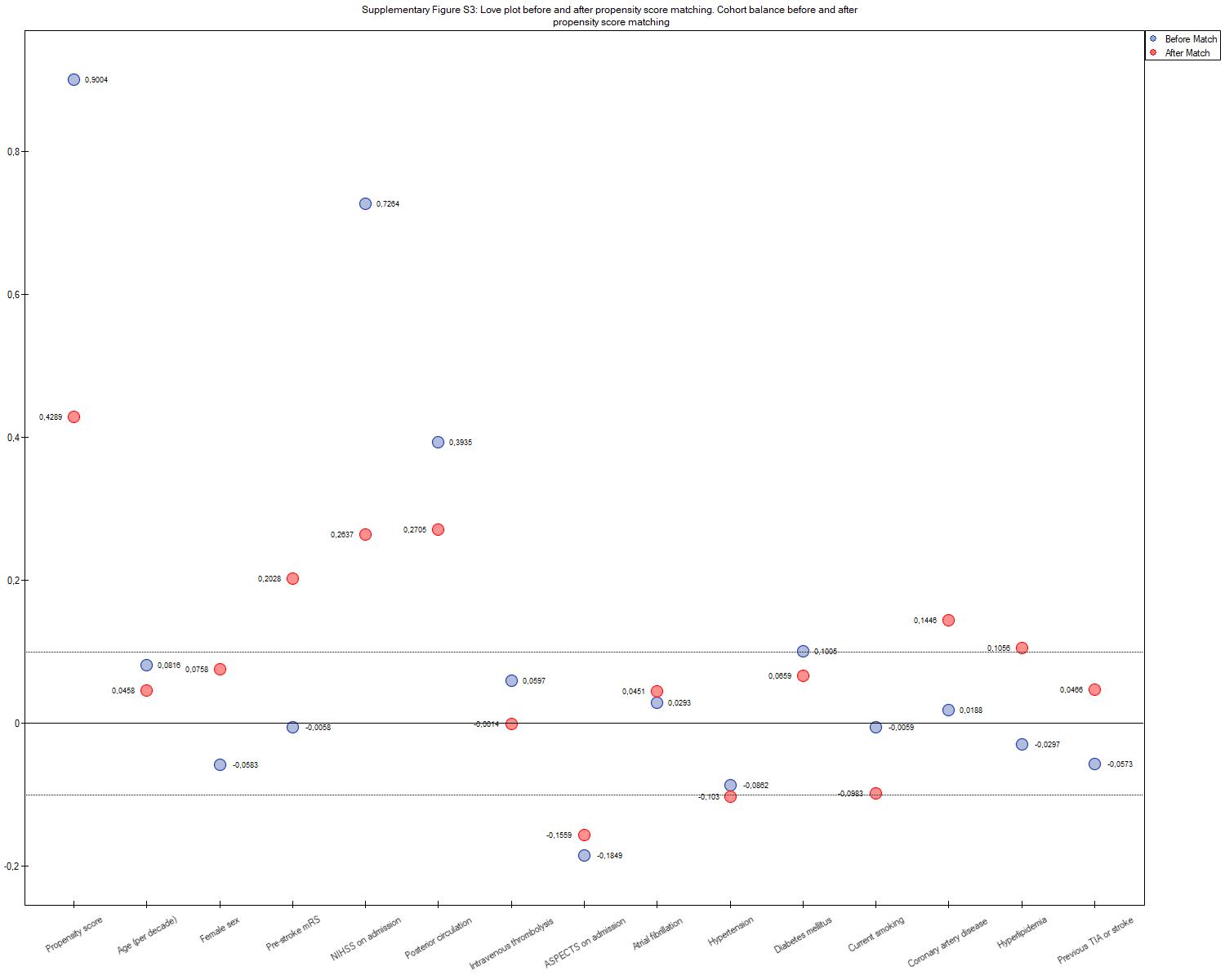

Supplement: Supplementary file 1 [file jcm-15-04993-s001.zip › Supplementary Figure S3 Love plot before and after propensity score matching. Cohort balance before and after propensity score matching.jpg]
